# Supplementary figures and images for: Transcriptional licensing is required for Pyrin inflammasome activation in human macrophages and bypassed by mutations causing familial Mediterranean fever
Source: PLoS Biol. 2022 Nov 7;20(11):e3001351. doi: 10.1371/journal.pbio.3001351 (PMC9671422; doi:10.1371/journal.pbio.3001351)

Figure 1c/ Fig S1c

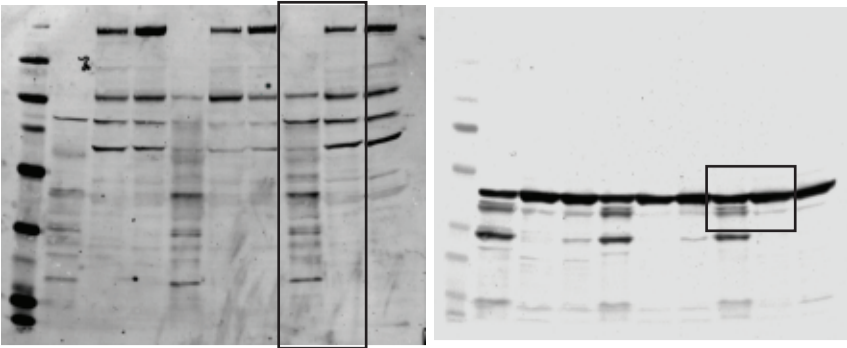

Figure 1e

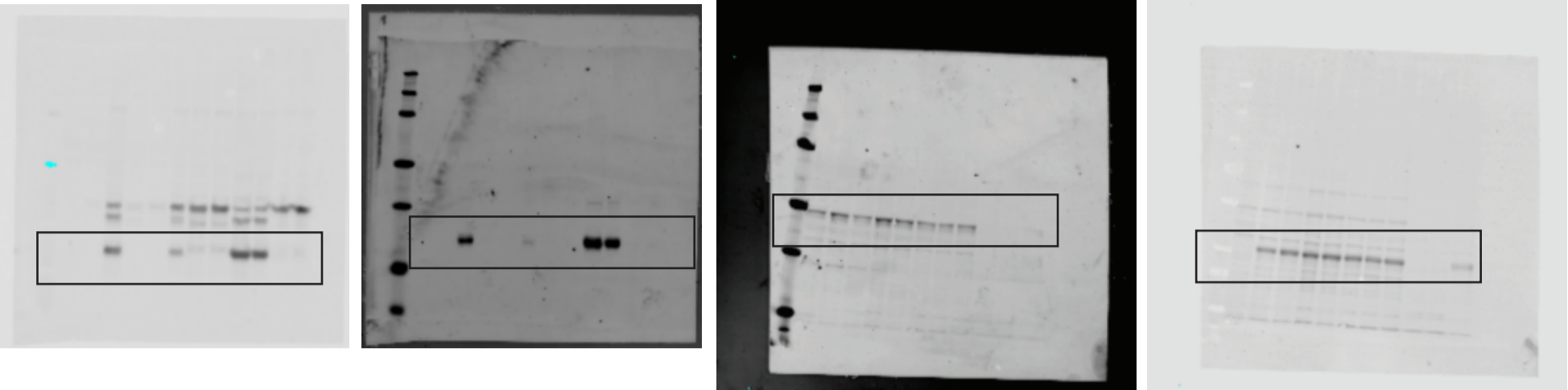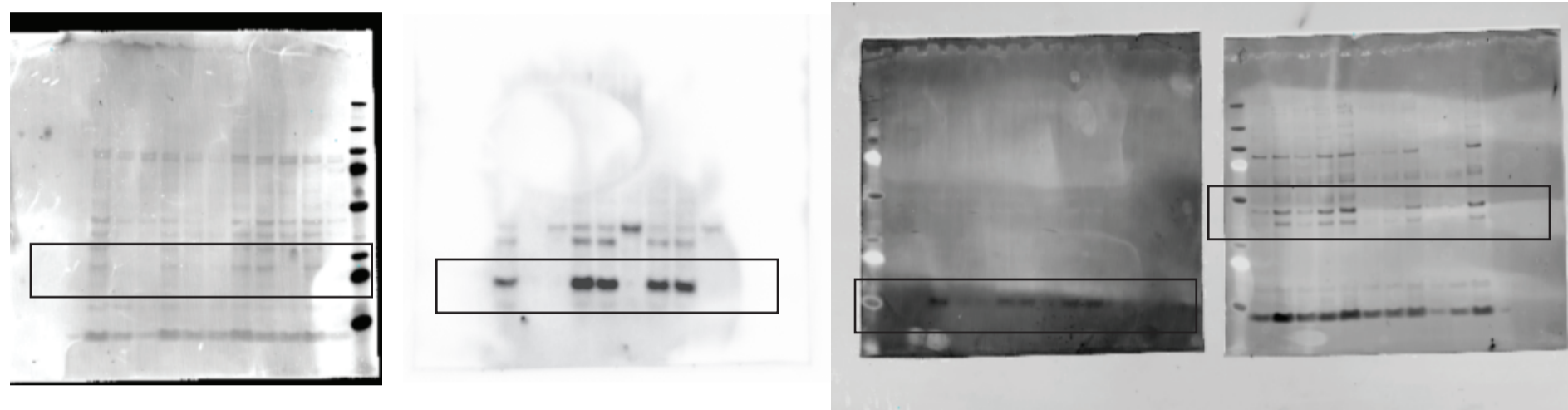

Figure S1a

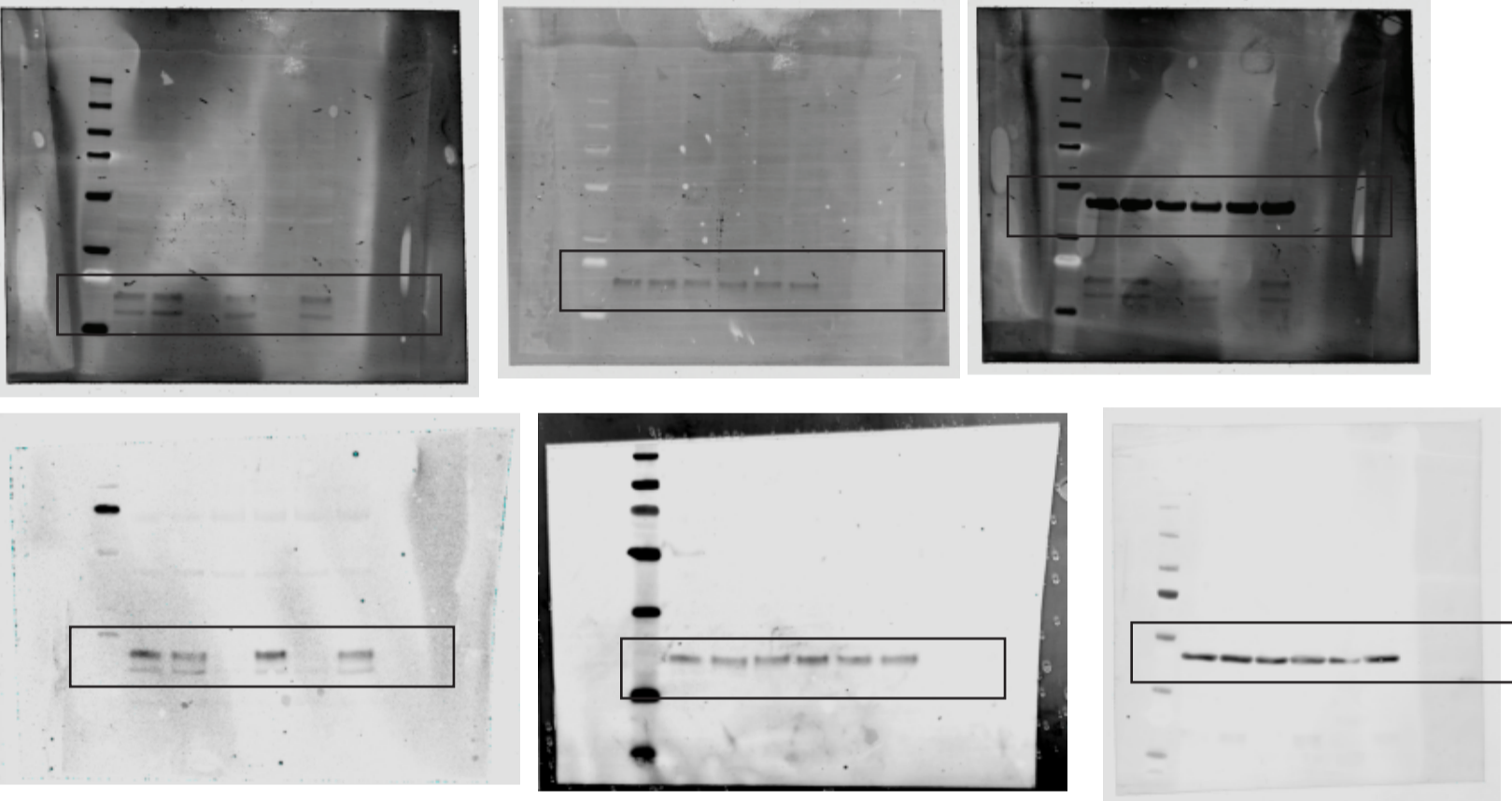

Figure S1b

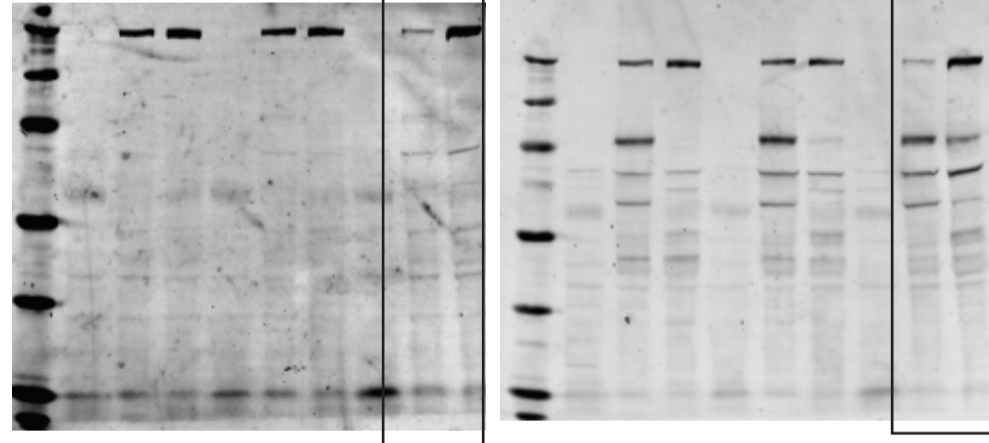

Fig S1c

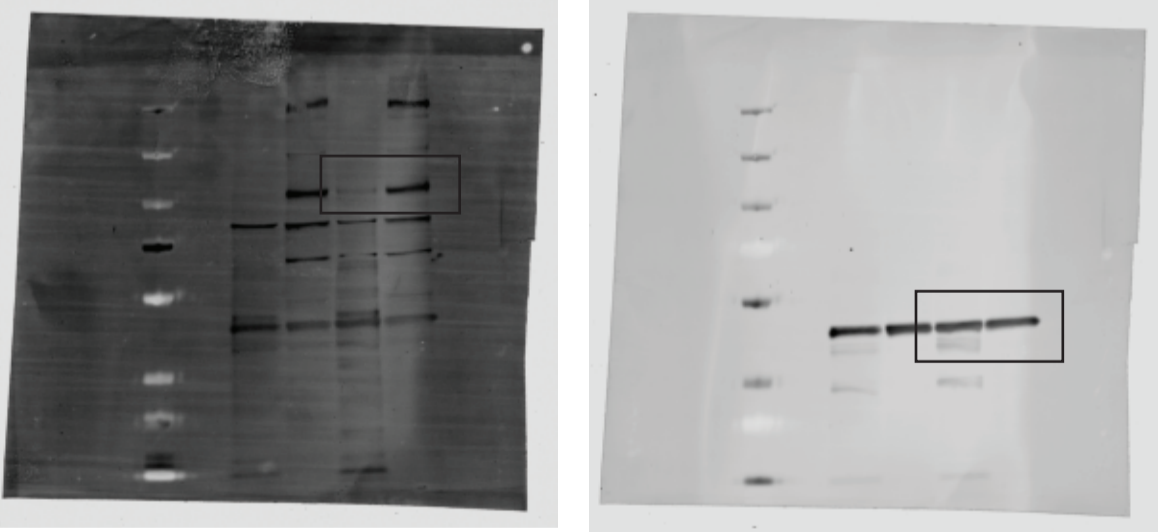

Figure 3

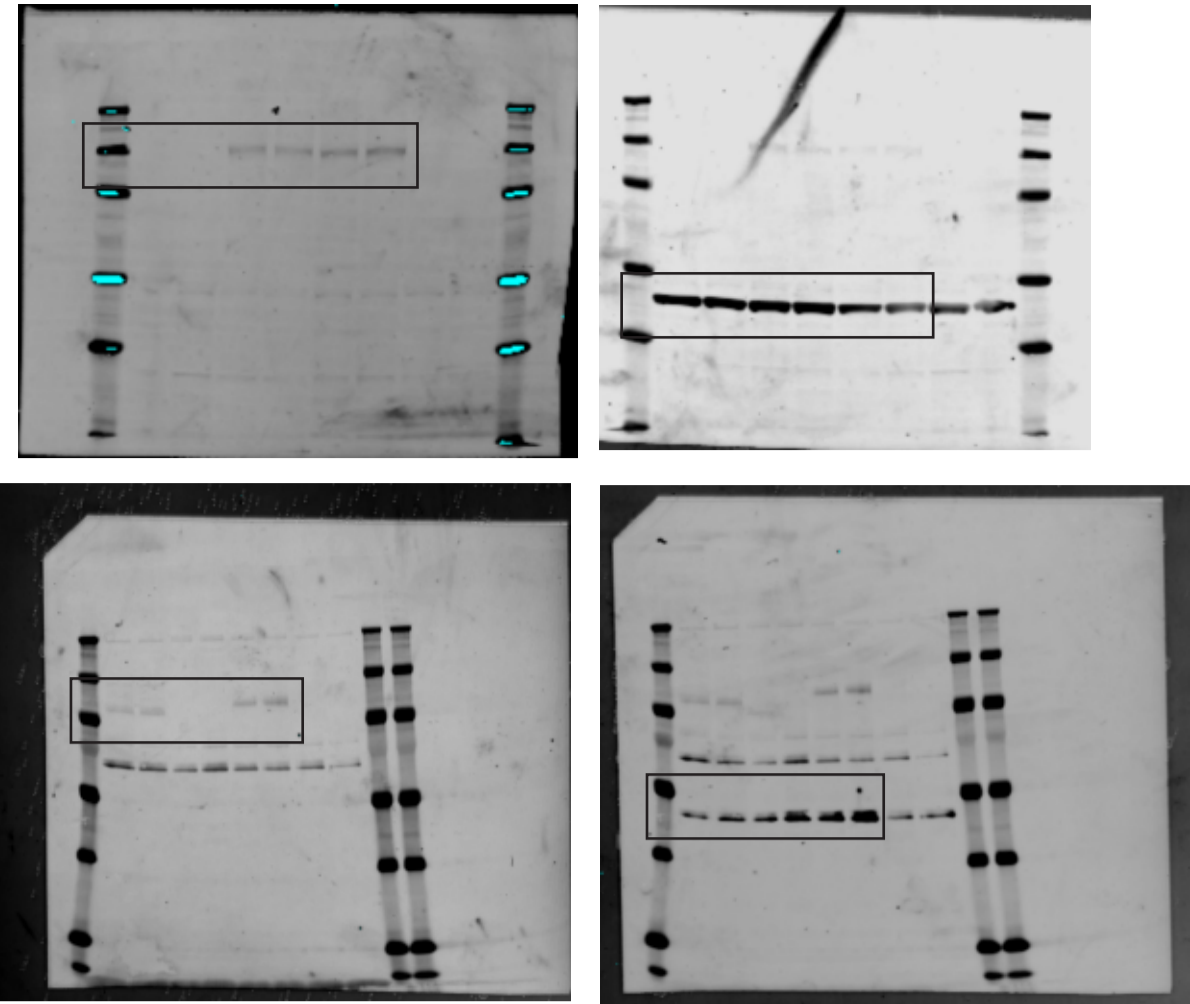

Figure 4

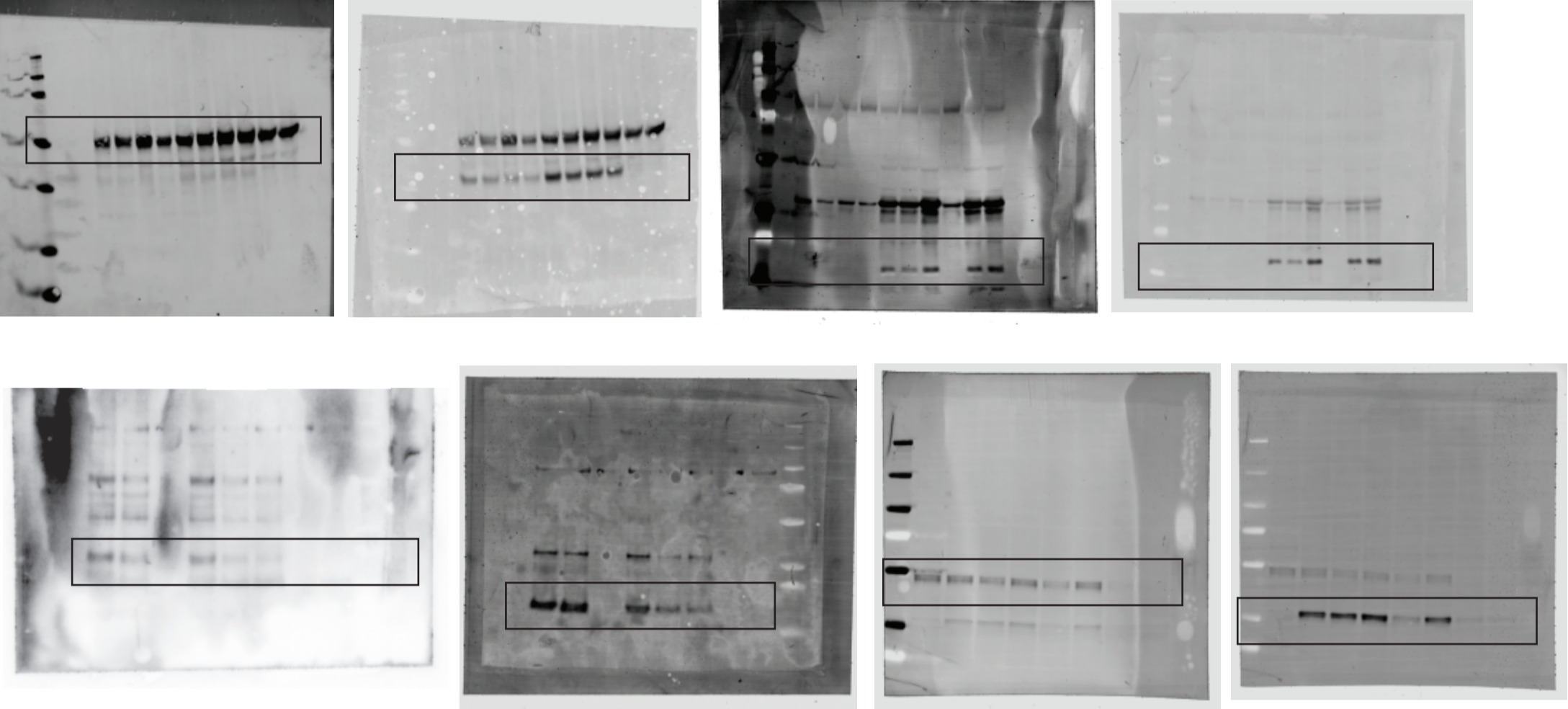

Figure 5

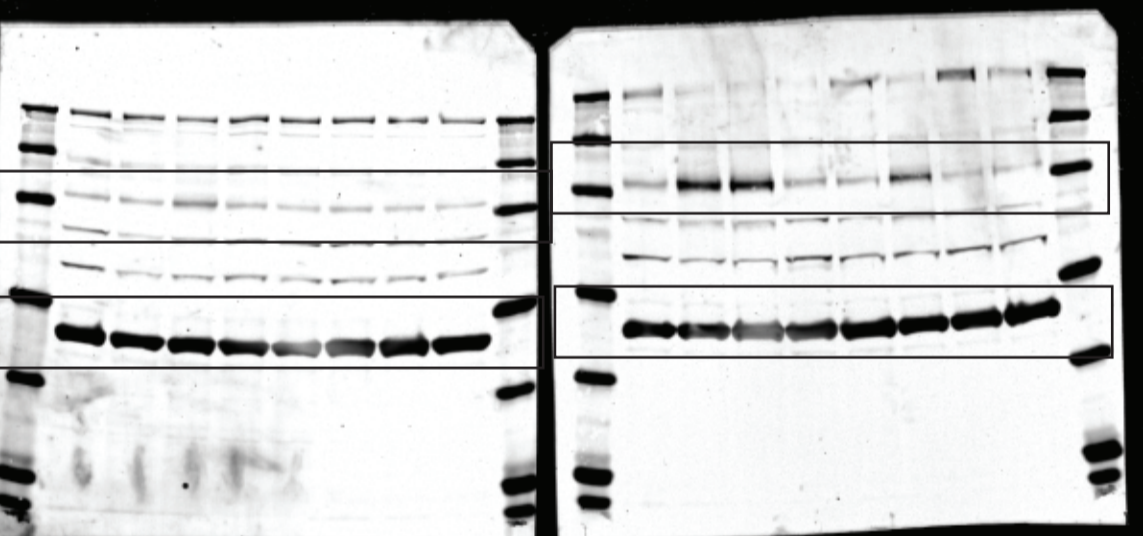

Figure 6

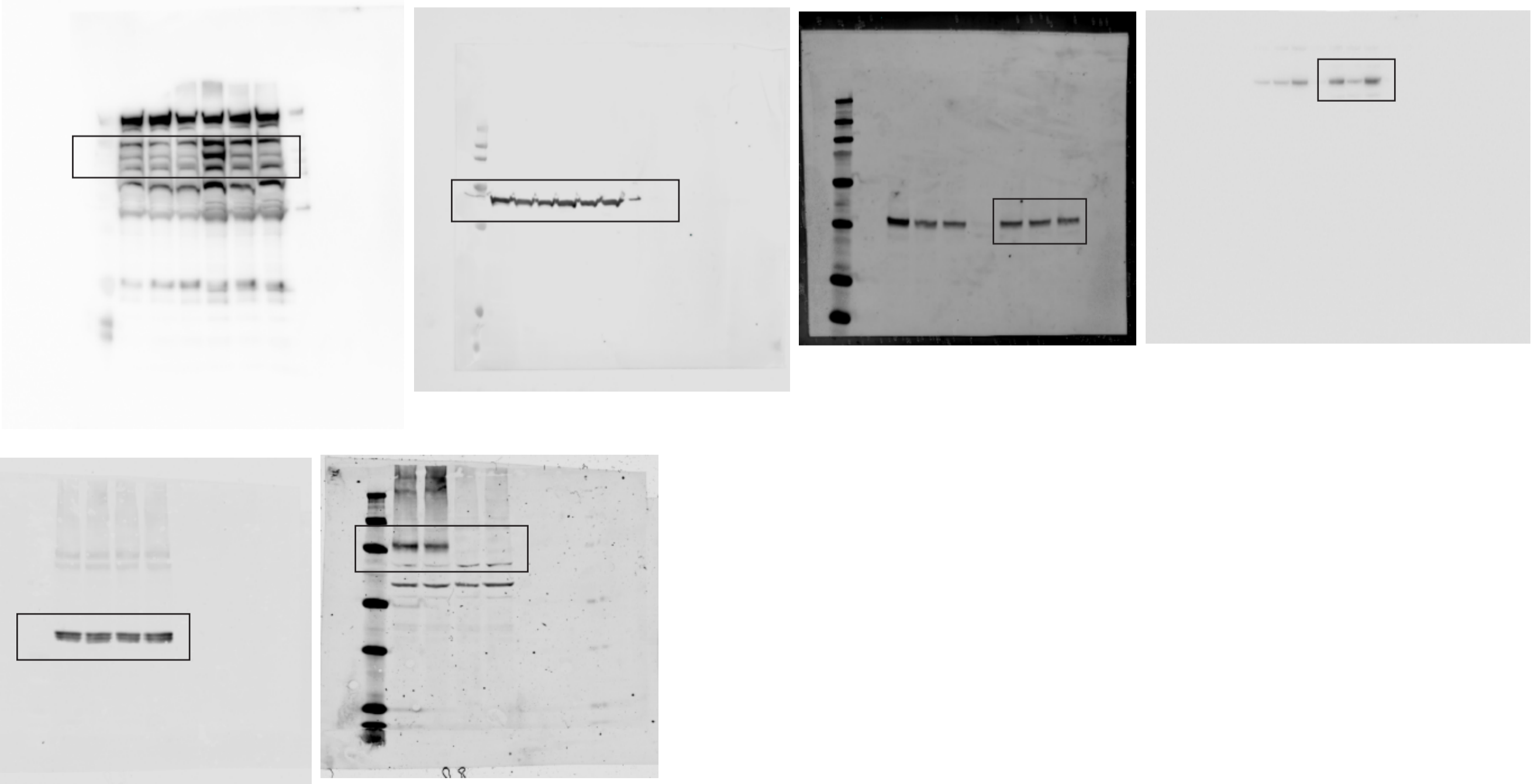

Figure S3

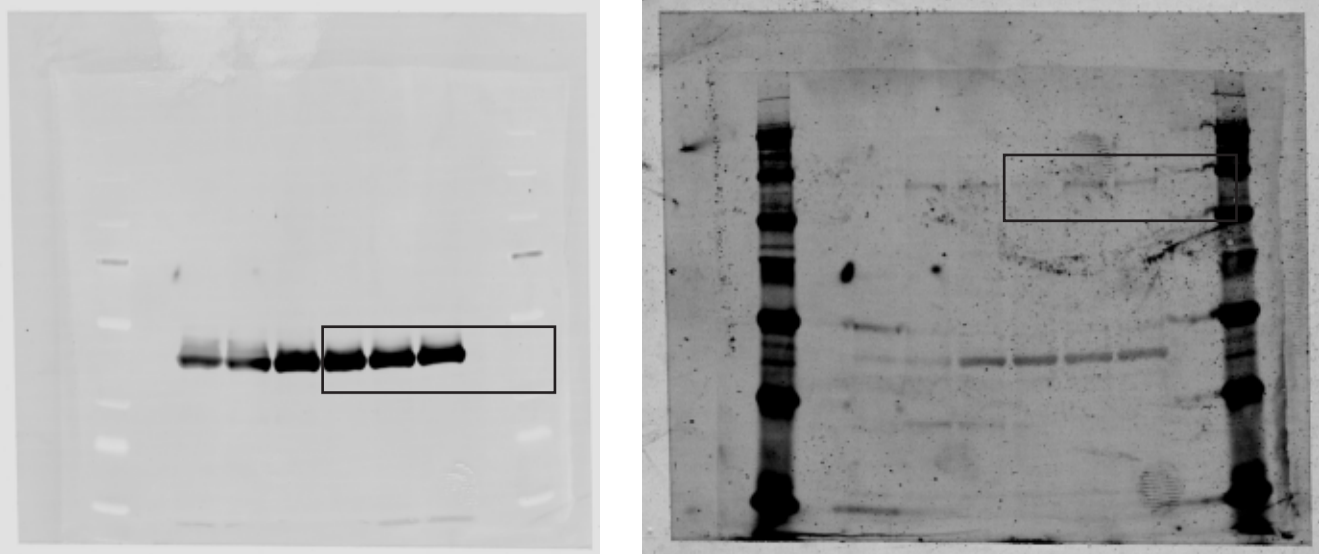

Figure 7

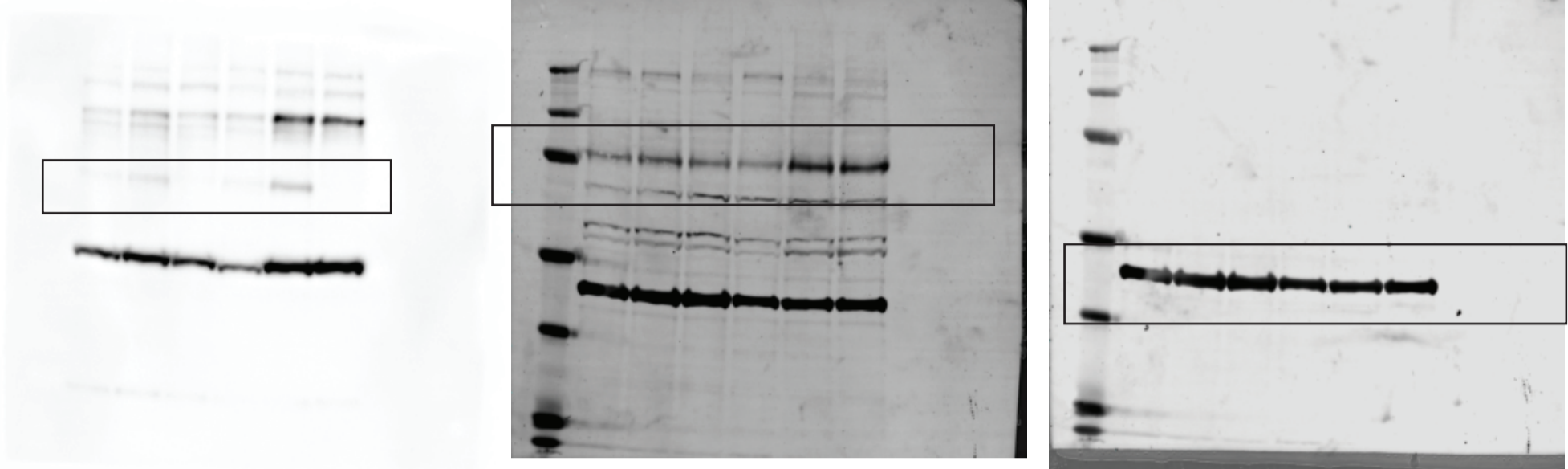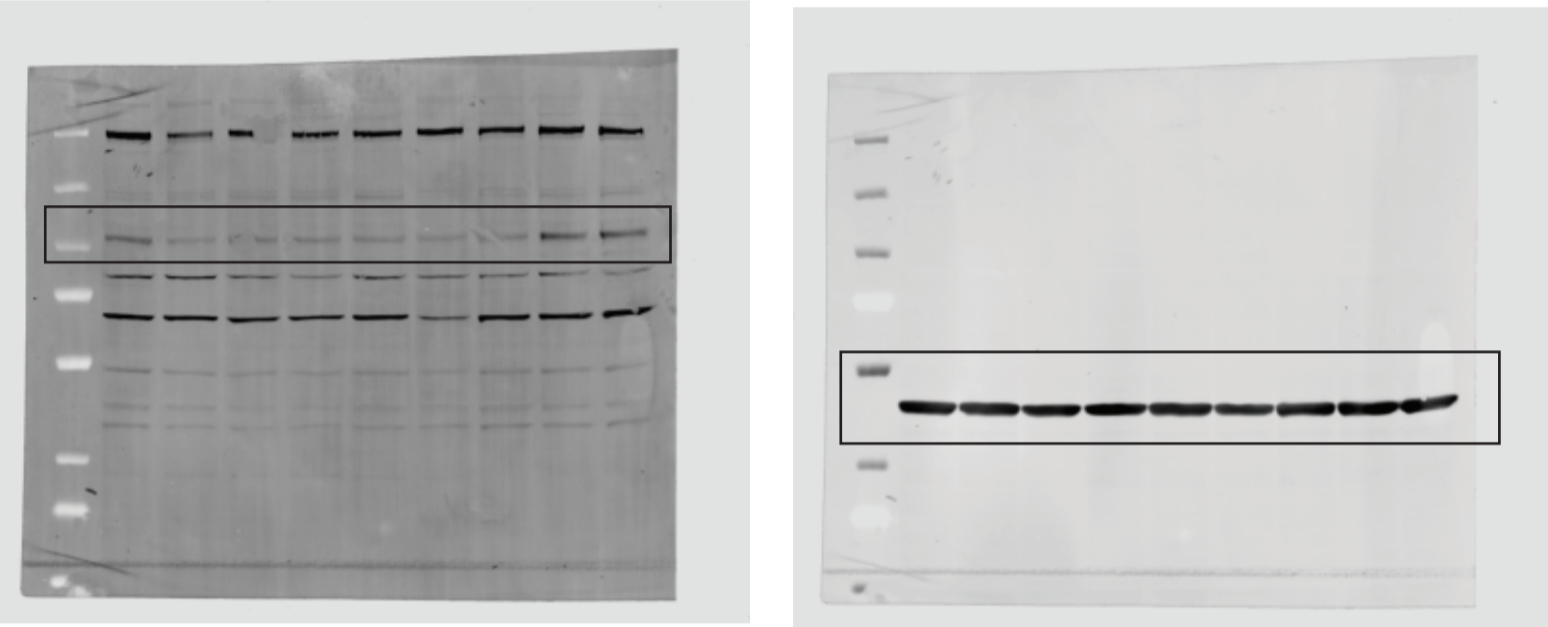

Supplement: S1 Raw Material — The excerpted portion of the immunoblot shown in the relevant figure is highlighted by a black box. (PDF) [file pbio.3001351.s002.pdf]

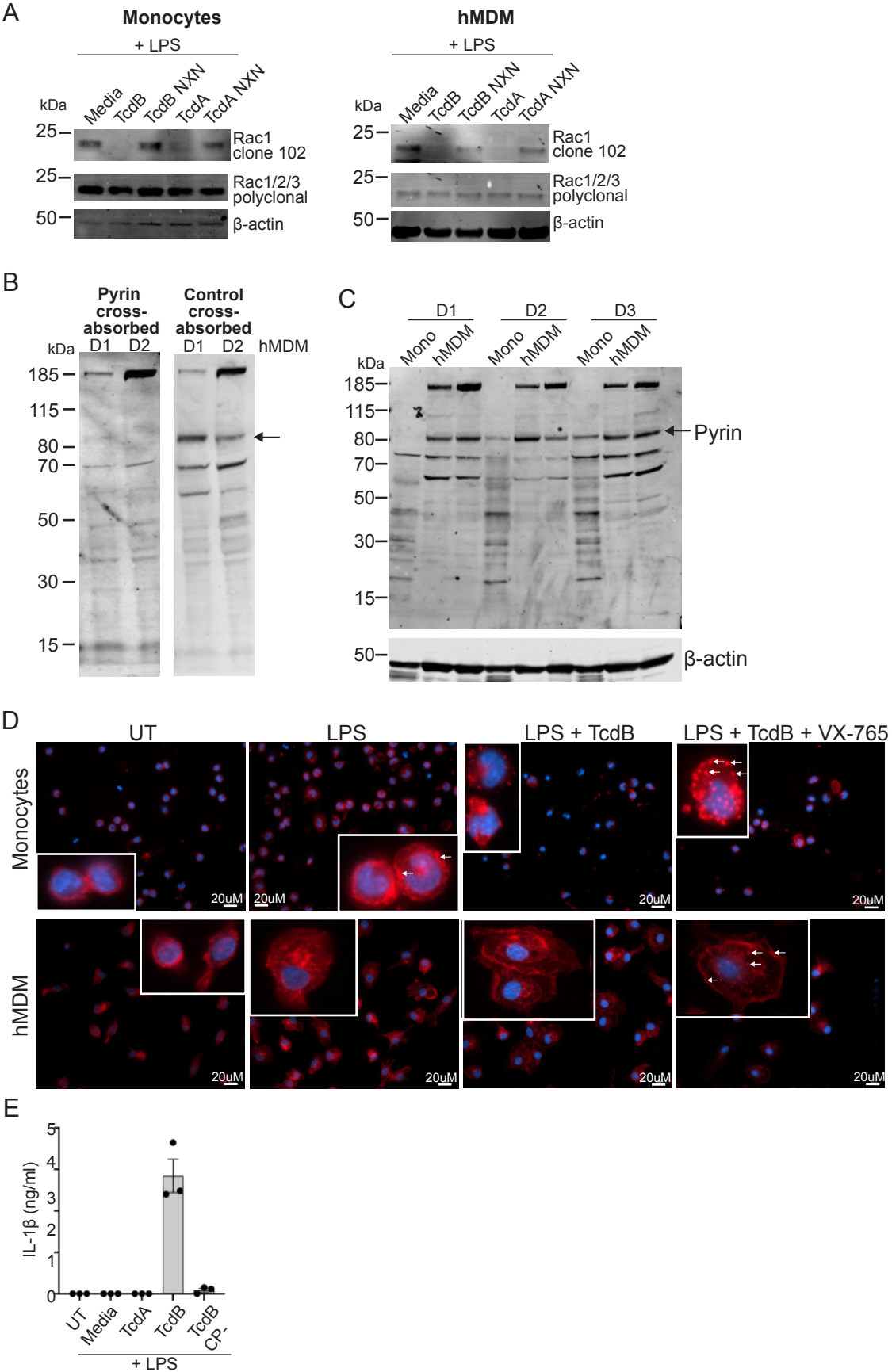

Supp. fig. 1

Supplement: S1 Fig — (A) Immunoblot of Rac glucosylation status in either monocytes or hMDM following treatment with the listed toxins (NXN variants lack glucosyltransferase activity). Representative of 3 experiments. (B) Immunoblot of hMDM lysate sequentially probed with the α-Pyrin antibody preabsorbed against HEKs transfected with Pyrin, then with the α-Pyrin antibody preabsorbed against those transfected then empty vector (control). (C) Pyrin expression in monocytes or hMDM from 3 different donors. (D) Actin staining following incubation of monocytes or macrophages with or without LPS and TcdB. Treated cells were fixed and stained with Phalloidin 647 to detect actin (red) or with DAPI to detect nuclei (blue). White arrows highlight the changes in actin distribution between the 2 conditions. Images are representative from 3 separate donors. (E) IL-1β release from LPS-primed hMDM differentiated for 7 days in M-CSF and stimulated with either TcdA or TcdB +/ CP-456,773. Mean and SEM shown for 3 independent donors, * p < 0.05, n.s. not significant. The underlying data can be found in the summary data file (S1 Data) in the tab S1E Fig. (PDF) [file pbio.3001351.s003.pdf]

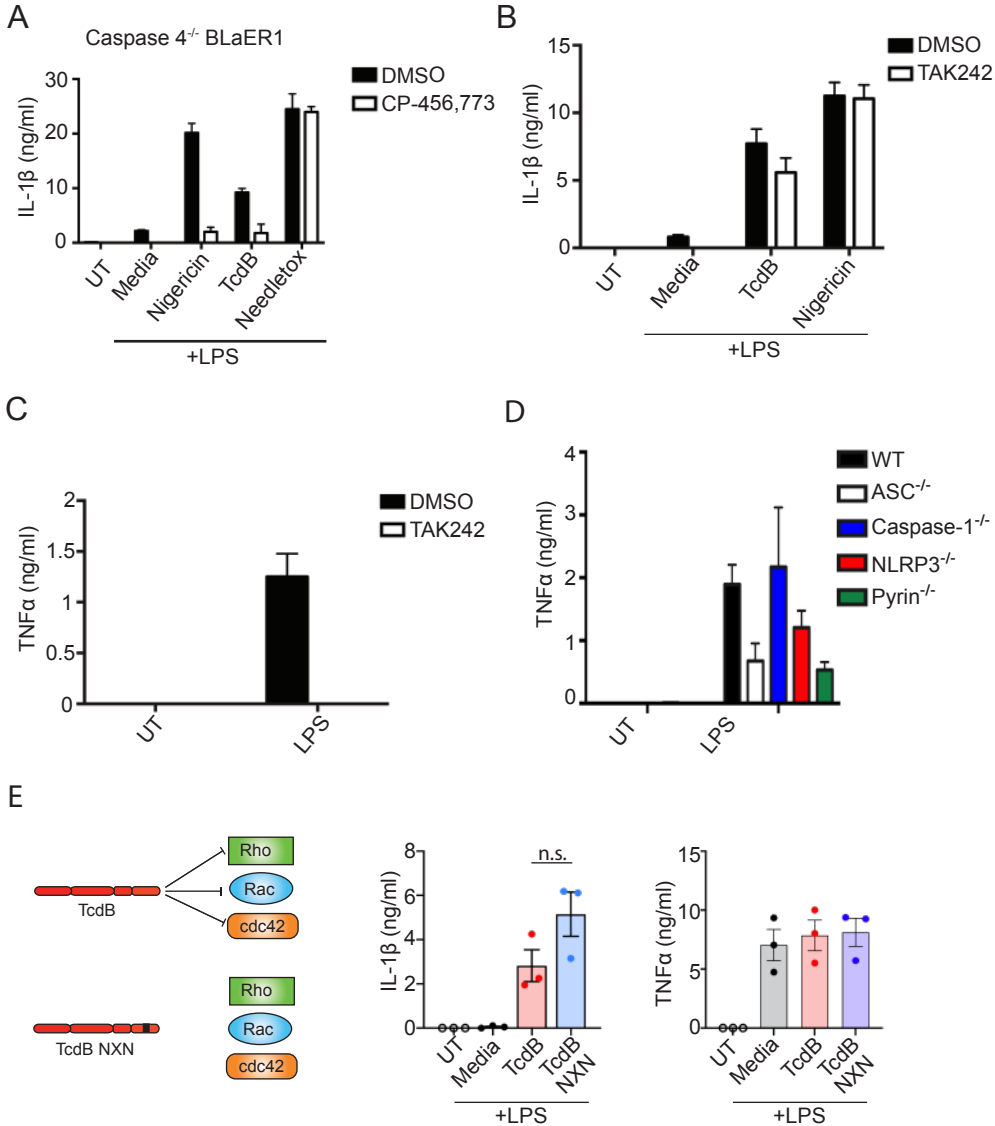

Fig. S2

Supplement: S2 Fig — (A) Differentiated caspase-4-deficient BLaER1 cells were stimulated as in Fig 3A. IL-1β was assessed from the harvested supernatants. (B) LPS-primed differentiated WT BLaER1 cells were preincubated with TAK242 (2 μM, 30 min) then activated with TcdB (20 ng/ml) or nigericin (8 μM) for 2 h. Harvested supernatant was assessed for IL-1β. (C) Differentiated WT BLaER1 cells were preincubated with TAK242 then stimulated with LPS for 4 h. TNFα was assessed from the supernatant. (D) TNFα was measured for THP-1 cells from Fig 3G. Mean and SEM shown for 3 independent experiments. (E) LPS-primed (10 ng/ml, 3 h) human macrophages were treated either TcdB or the TcdB NXN mutant lacking glucosyltrasferase activity (20 ng/ml, 2.5 h). Supernatant was harvested and assessed for IL-1β or TNFα. For (A-C), the mean and SD of 3 technical replicates shown, representative of 3 independent experiments. For (D) and (E), the mean and SEM shown for 3 independent experiments. * p < 0.05, n.s. not significant. The underlying data can be found in the summary data file (S1 Data) in the tab S2B–S2E Fig. (PDF) [file pbio.3001351.s004.pdf]

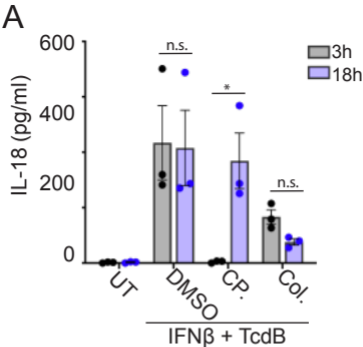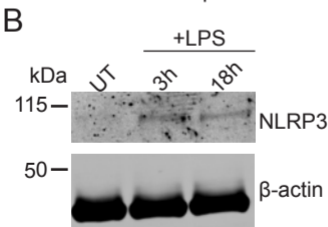

**Fig. S3**

Supplement: S3 Fig — (A) IFN-β (5,000 U/ml, 3 or 18 h) primed hMDM preincubated with compounds as previous and stimulated with TcdB (20 ng/ml) for 2.5 h. The supernatant was harvested and assessed for IL-18 release. (B) NLRP3 expression in either untreated hMDM or incubated with LPS for 3 h or 18 h as assessed by immunoblot, representative of 2 independent donors. Mean and SEM shown for 3 independent donors for cytokine release, *p < 0.05, n.s. not significant. The underlying data can be found in the summary data file (S1 Data) in the tab S3A Fig. (PDF) [file pbio.3001351.s005.pdf]
