# Supplementary material for: Transcriptional licensing is required for Pyrin inflammasome activation in human macrophages and bypassed by mutations causing familial Mediterranean fever
Source: PLoS Biol. 2022 Nov 7;20(11):e3001351. doi: 10.1371/journal.pbio.3001351 (PMC9671422; doi:10.1371/journal.pbio.3001351)
Supplement: Attachment — Submitted filename: TcdB-Pyrin_manuscript_response_to_reviewers_final.docx [file pbio.3001351.s006.docx]

**Point-by-point response (PBIOLOGY-D-21-01608R1)**

We thank the editor and reviewers for their thoughtful review and constructive comments, which have led to an extensive revision of the original manuscript. We believe that these new experiments both reinforce the initial findings of the study. Importantly we have now included new human translational experiments using Familial Mediterranean Fever (FMF) patient material to provide insights into the mechanism holding Pyrin inactive in human cells and how this is perturbed by mutations causing FMF. The reviewers’ comments were carefully considered and addressed below, and changes in the text prompted by the reviewers’ comments are in red.

Together, our previous and new supportive findings have strengthened the revised manuscript

which support the following concepts:

- **The Pyrin inflammasome cannot be activated in human macrophages under basal conditions by *C.difficile* toxins. We have now confirmed this with an addition Pyrin activator, the bile acid analogue 11-oxo-12*S*-hydroxylithocholic acid methyl ester (BAA-473).**
- **The Pyrin inflammasome response in human macrophages requires prolonged exposure to LPS or type I interferons. This has been similarly confirmed with the bile acid analogue 11-oxo-12*S*-hydroxylithocholic acid methyl ester (BAA-473).**
- **The B30.2 domain of Pyrin is the domain primarily required to prevent Pyrin inflammasome activation in human macrophages.**
- **Mutations in the B30.2 domain that cause Familial Mediterranean Fever enable Pyrin inflammasome activation in human macrophages.**
- **In the absence of Pyrin activation, Toxin B from *C. difficile* activates the NLRP3 inflammasome, demonstrating redundancy at the level of inflammasomes in sensing this toxin.**

**Reviewers’ comments**

**Reviewer 1**

Mangan and colleagues described the interesting finding that in human macrophages (hMDMs) TcdB activates the NLRP3 inflammasome while both TcdA and TcdB fail to activate pyrin inflammasome while they do so in primary human monocytes. This is an important description since these two toxins are the main tools to activate the Pyrin inflammasome.

A catalytic mutant of TcdB, unable to modify RhoA (the key signal to trigger pyrin inflammasome activation) triggers NLRP3 activation. This is highly unexpected, nicely shown with the recombinant catalytic mutant TcdB protein! But not the focus of this manuscript.

Long LPS (or IFN)  treatment induces pyrin expression and triggers TcdA/TcdB inflammasome responses that are pyrin-dependent. Several experimental systems are used to demonstrate that the transcriptional increase in Pyrin can restore TcdB-mediated pyrin inflammasome activation. Transcription regulation is clearly contributing to this regulation although other data in this manuscript suggest that the regulation is likely more complex.

Overall, while the mechanistic underlying the regulation of pyrin and the mechanisms associated with TcdB-mediated activation of NLRP3 remain to be deciphered, this paper is important to set the bases for future works aiming at understanding inflammasome regulation in healthy donors during C. difficile and potentially Yersinia or Burkholderia infections, and in patients with autoinflammatory diseases.

The knowledge on the Pyrin inflammasome is lagging far behind other inflammasomes and it is important for the field to gain novel insights onto this inflammasome complex which is highly relevant for infections and autoinflammatory syndromes. At this stage a number of experiments are required to support in a robust manner the authors conclusions.

Main comments:

1-The most trivial explanation: difference in expression levels between primary monocyte and hMDM should be better established. Indeed, Pyrin has been described to have reduced expression level in hMDMs compared to monocytes both at the transcriptional and protein level (see Fig 2 in Gavrilin MA et al JI 2009).
The data is based here on one Western Blot  panel with a single donor (Fig. 1C). Furthermore, Pyrin is migrating under 80kDa in this panel while it should be above. Pyrin can have multiple degradation bands and you should show the full Western blot (see Chae JJ et al Blood 2008 or your own WB Fig. 5D).

This WB in Fig 1C thus needs to be repeated by showing the band corresponding to the Full Length protein and in three donors to robustly rule out that the expression level of the FL pyrin is not the trivial explanation. qRT-PCR must also be performed to validate (or not) the findings at the transcript level.

- We appreciate the reviewer’s comment and apologise that the immunoblots were inadvertently moved relative to the MW markers for a few of the immunoblots during the final editing process. This has been corrected now. We have included additional data to answer the reviewer’s comments
  - We have added immunoblots of hMDM lysate probed with Pyrin antibody pre-absorbed against the lysate of pyrin or vector alone transfected HEK that we had performed previously to identify which bands of the band(s) are specific for Pyrin. We find that only one band was entirely not evident after pre-absorption against the Pyrin-containing lysates, which was just above 80kDa.
  - The qPCR was not performed as we thought it was more important to demonstrate equivalent protein levels, and so have done further work to validate our system in that direction instead. We were also concerned that analysis of Pyrin transcript may be misleading as a measure of expression, as Pyrin expression is also controlled on a transcriptional level through nonsense-mediated decay (ref: Grandemange et al. Hum Mol Genet. 2009 Dec 15;18(24):4746-55)
  - We have updated the main figure to show the entire blot for one donor and added the blot for four donors in the supplementary figure.
  - The text was edited starting at line 254.

hMDMs are usually obtained after 7 (sometimes 10 days) of monocyte differentiation. If I am correct, you differentiate monocytes during 3 days. It would be important to validate the key findings (NLRP3-dependent TcdB response and lack of TcdA response) in hMDMs differentiated with a standard protocol.

- We differentiate the monocytes for three days prior to harvesting, after which they are plated and incubated overnight in the presence of 50U/ml M-CSF. So the total differentiation time is around four days total. It is true that previously 7-10 days for monocyte to macrophage differentiation was used. However, this was based on phenotypic changes in culture. Recently, a study analysing transcriptomes of monocytes and macrophages has demonstrated that differentiation of monocytes to macrophages occurs after three days (Ref: Transcription based network analysis reveal a spectrum model of human macrophage activation. Xue et al. Immunity 2014). We use this exact protocol in our study.
- We have added a data panel (Fig S1e) using macrophages differentiated for 7 days in M-CSF, which shows the same result as those generated in 4 days.

2-The functionality of endogenous pyrin in BlaER1 cells and THP1 monocytes should be demonstrated if you would like to draw conclusions on the lack of role for endogenous pyrin in  BlaER1 macrophages and THP1 macrophages. For WT BlaER1, Pyrin may be truncated since it migrates at a lower MW than upon complementation on the WB Fig 2E. Furthermore, pyrin up-regulation by LPS seems defective (please indicate the priming time and LPS concentration). Can you generate BlaER1 in a monocyte like differentiation to check the functionality of pyrin? Otherwise this limit should be clearly mentioned.

- The complemented Pyrin has a flag tag, which explains why it runs slightly higher. This is now detailed in the materials and methods, and the figure and figure legend were also updated to include this information.
- The figure legend has been updated to include the information for the LPS priming. This was 100ng/ml for 3h, the same as used for the inflammasome stimulation experiment.
- Interestingly, the BLaER1 cell line was initially suggested to model monocytes rather than macrophages, but our data indicate that they behave more as macrophages at least in relation to Pyrin. Unfortunately, they can only be differentiated in one way and so cannot be made more “monocyte-like”. Therefore, we have updated the text to include this limitation.

Same question for THP-1. Did you check the functionality of THP-1 pyrin inflammasome in THP-1 "monocyte" (i.e. in the absence of PMA and in response to TcdA and TcdB). It is described to be functional, but you should check it in your experimental system.

- We stimulated non-PMA treated THP1 cells with either TcdB, TcdA or BAA-473 and assessed Pyrin activation to address this comment. However, we found that these cells did not consistently secrete IL-1b in response to any of these triggers. In general, we found that the monocytic THP-1 cells were less responsive to all signals than the PMA differentiated cells. We have updated the manuscript to include this caveat as well.

3- The manuscript ends on an unfinished Figure 6. Authors have investigated the step1 (S242 dephosphorylation which seems to be functional in hMDM), they thus need to investigate whether FMF patient hMDMs defective in the step 2 regulation would be responsive to TcdA as suggested by Shiba et al (JACI 2019) and whether TcdB responses would be blocked by colchicine, MCC950 or neither of each. Clearly, these experiments are missing. 
The legend in Figure 6 "and is disrupted by FMF mutations" suggests that the authors have at least thought about this experiment which must be performed in primary cells from FMF patients or at least using reconstitution of BlaERcells and/or THP-1 cells with FMF MEFV alleles.

- We thank the reviewer for highlighting this problem. To address this question, we have included new data assessing Pyrin inflammasome activation in Pyrin KO THP1 cells reconstituted with either human Pyrin or murine Pyrin (Fig 6b-d). We have also tested Pyrin activation in monocytes or macrophages isolated or derived from FMF patients and compared them to non-FMF controls. This data is also included in Figure 6.

4- We have learned from NLRP3 that inflammasome regulation is highly complex. While I have no doubt that MEFV up-regulation contributes to pyrin responses, this is likely not the only regulation as suggested by Fig 6 and Fig 1C. To strengthen the role of LPS treatment per se vs. up-regulation, Fig 5F should be performed without LPS treatment looking either at IL-18 or at ASC specks.

- We thank the reviewer for the suggestion. The data addressing this issue is presented in Fig 5F-H. These show that Pyrin is overexpressed in these cells and enables a Pyrin response in the absence of LPS as measured by LDH release. We have also included new data in Fig 6 where we reconstituted a Pyrin KO THP1 cell line with Pyrin and found this enables a Pyrin inflammasome response. However, given that these cells express substantially more Pyrin (more so than hMDM incubated with LPS for 18h), we have changed and altered the title and the text to reflect that effects other than transcriptional regulation might also be involved.

Fig 5F: blot is missing and should be shown. Do you really have MEFV overexpression. Based on Fig. 2E (overexpression in the MEFVko background), this is not obvious.

- We have included the immunoblot suggested in Fig 5F

5- The discussion is extensive which is nice to see and read. One paper is missing and should be discussed Shiba T et al in JACI 2019 since it illustrates another difference between monocytes and  hMDMs: the fact that in hMDMs from FMF patients, TcdA-mediated pyrin inflammasome activation is blocked by colchicine while it is not in monocytes. Furthermore, in this paper a short priming (2h) with a large dose of LPS (1ug/ml) is sufficient to  promote TcdA -mediated pyrin inflammasome activation. Finally, looking carefully at the data, one might observe that hMDMs from healthy donors do not respond to TcdA while hMDMs from FMF patients do.

- We thank the reviewer for highlighting this study - it is indeed interesting to see the differences in Pyrin activation between the macrophages with and without the FMF mutation. We have included a comment on this in the discussion (line 1055)

6-Numerous inaccuracies or overstatements (see minor comments).

Minor comments
1-I suggest to edit the title: demonstrating the presence of a novel regulatory mechanism. (The demonstration of the regulatory mechanism is not complete at the moment).

The title has been updated.

2-Clostridim is the old nomenclature, please edit throughout.

This has been edited.

3-Line 55 and throughout: Please italicize gene name.

We have edited this as suggested.

4-Line 68: the redundancy is limited to one toxin.

This has been edited.

5-Line 75: pyrin oligomerization upon activation remains to be demonstrated (the current literature indicates that pyrin is a trimer at steady state).

This has been edited.

6-Line 101: the requirement for microtubule dynamics (as opposed to microtubule polymerization/integrity) is controversial. It is based on paclitaxel (Taxol) shown to inhibit pyrin inflammasome activation in Gao et al but to be inefficient in Van Gorp et al and in Magnotti et al**.**

The sentence has been edited to specify the susceptibility to microtubule destabilising compounds

7-Line 148: these results suggest that pyrin is held in an inactive state (instead of demonstrate).

This has been edited.

8-Line 216: It would be interesting to assess whether RhoA is inhibited in hMDM by TcdA (and TcdB). The commercially available Rho A G-LISA activation assay could be used (although Fig 6A suggests Rho A is indeed inhibited).

We agree, though we believe that the data provided in figure 6A, as well as the effect on actin observed in Supplementary figure 1 is sufficient to suggest that RhoA is inhibited.

9-Line 238: Since microtubules have been shown to contribute to NLRP3 activation (Magupalli VG et al Science 2020), the efficacy of colchicine suggests the dependence on pyrin but does not demonstrate it.

We are aware of this study, though in our hands we did not observe any effect of colchicine on NLRP3 activation, whilst CP-456,773 was effective in every case. Indeed, we never observed an inflammasome response that was both CP-456,773 and colchicine dependent. Notably, in the Magupalli et al. they needed a higher concentration of colchicine to inhibit NLRP3 compared to Pyrin, suggesting that the compound effects these two sensors differently. Taken together, we believe that colchicine at the concentration at which it is used is specific and so does demonstrate a dependence on Pyrin.

10-Line 245: With the quality of the images at the moment, it is hard to conclude anything. We do see the cell death in monocytes, but the actin disruption in the presence of VX-765 is not obvious.

We thank the reviewer for highlighting this and agree in the state in which they were presented the quality was insufficient to determine actin disruption. This was due to condensing the images for generation of the pdf, and we apologise that this was not caught prior to submission. We have increased the quality of the image and increased the level of the actin staining to make the interpretation of the images possible.

11-Line 394: change the title of figure 3: from "Pyrin is the responding sensor" to "NLRP3 is not the sensor". This is what is demonstrated in Fig 3.

This has been altered.

12-Line 445, 453, 485, 532 : Pam3CSK4

This has been edited.

13-Fig 4B: please edit the y-axis to MEFV/IL1b transcript or mRNA

This has been edited.

14-Line 450: ISRE

This has been edited.

15-Line 513: "demonstrating that increased expression is linked to pyrin reactivation."
This is an overstatement. At best, you have a correlation. Plus, how do you explain that you have lost the NLRP3 response?

We regard linked and correlated as synonymous but can understand others may not agree, and so have updated the text as recommended. Regarding the loss of NLRP3 response to TcdB, we have no clear explanation. NLRP3 is expressed at equal amounts after 3h or 18h LPS priming can still be activated by nigericin in hMDM primed with LPS for 18h. It is possible that prolonged exposure to LPS/IFN changes the pathway perturbed by TcdB to trigger NLRP3 activation, rendering NLRP3 specifically insensitive to activation by TcdB. Alternatively, Pyrin activation will almost certainly occur faster as it is likely to be more direct, and so any macrophage that takes up enough toxin to activate an inflammasome activates Pyrin preferentially.

16-Line 529: I am not sure you can compare a pool of untreated cells with a pool of siRNA-treated cells. You could have a full knock-down in 90% of the cells.

We take the point of the reviewer, but assessing knock down in this way is standard practice in the literature. We specifically used a short time frame following transfection that should prevent translation of new protein whilst leaving the already translated protein intact - indeed the level of Pyrin expression is similar between cells with no LPS treatment and those with LPS in the presence of the Pyrin-specific siRNA, indicating that the siRNA likely does not decrease Pyrin but rather prevents the LPS driven increase. Ideally, we could support this with a FACS based readout, but we do not know of any antibody that can be used to detect Pyrin by immunocytochemistry or flow cytometry.

17-Line 592: to my knowledge, no endogenous molecules have been demonstrated to inhibit RhoA signaling pathway and trigger pyrin activation.

We thank the reviewer for highlighting this error and have removed it from the text.
18-Line 594: change "multiple" to "two".

We simply state here that understanding Pyrin activation in different cell types is important, rather than referring directly to the work in the manuscript.
19-Line 619: please double check Centola et al (ref 24) for the use of PBMCs vs. monocytes and Pam3CSK4 vs. LPS & TNF. Similarly, Pyrin is induced by F. novicida infection, a very poor TLR4 activator (Gavrilin A et al, JI 2009).

We thank the reviewer for highlighting this error and have changed the text to be specific for monocytes. The comment on *F. novicida* has been removed as part of the editing process to focus the discussion. We are aware of the results from *F. novicida*, but in spite of being a poor TLR4 activator, it stimulates robust IFN production, which may account for the increase in Pyrin expression in this case.

20-line 623: to inhibited

This has been edited.

21-line 625: could you add a reference for inflammatory signals regulating AIM2 inflammasomes.

This has been edited.

22-line 631: Gram-negative

This has been edited.

23-line 633: novidica

This has been edited.

24-line 634: "Pyrin is the responding sensor to F. novicida in hMDM [25]." This is controversial see Lagrange et al Nat Com 2018 and Mitra S et al Plos 1, 2018.

We have updated the manuscript to include the additional references and a note on the differences between the studies.

25-line 637: "IL1b blocking therapy is only somewhat successful in preventing the disease [26]". 
This sentence conveys a false message. IL1b blocking therapy is successful (at least to my knowledge and according to ref 26).

This sentence has been removed in the redraft of the discussion.

26-Line 639 that stimulates.

Not has not been edited, as we believe the current phrasing is correct.

27-line 644: comparible

This has been edited.

28-line 652: van Gorp et al did not demonstrate a requirement of the B30.2 domain for the colchicine sensitive pyrin activation. They demonstrated that FMF-associated point mutations in the B30.2 domain abolish colchicine-mediated inhibition, which is not the same (as described in the intro, mouse pyrin lacks B30.2 but its activation can be blocked by colchicine).

We thank the reviewer for highlighting this error and have changed the text to reflect this.

29-line 668: My understanding was that AIM2 signaling was deficient in both human monocytes and macrophages not that there was a difference between monocytes and macrophages.

We have removed the text as we determined that there was insufficient evidence for this statement - Thus far the deficiency in AIM2 signalling has only been demonstrated in monocytes. The response in primary human macrophages has not been assessed yet (to our knowledge) whilst PMA-differentiated THP1 cells respond in an AIM2-dependent manner.

30-line 681: endogenous bile salts do not activate pyrin. Ref 29 describes potential bile acid metabolites which might be generated by the sequential action of two bacterial enzymes.

We thank the reviewer for highlighting this error and have altered this in the text.

31-line 689: here you could cite the Aim2 work by Gaidt and colleagues.

We have not included the citation here as we believe the other references cover the point sufficiently.

32-line 693: I don't get the M-CSF point. Pyrin is highly expressed in neutrophils. 3 days of M-CSF treatment (Fig. 1C) does not modify Pyrin expression levels.

We have removed this from the text, we agree with the reviewer, in the context it does not make sense.


**Reviewer #2:** 
The manuscript by Mangan and colleagues entitled "Clostridium difficile Toxin B activates the NLRP3 inflammasome in human macrophages, demonstrating a novel regulatory mechanism for the Pyrin inflammasome" investigated the sensing of Toxin B by inflammasomes in human monocytes and macrophages. This is a very interesting topic and a timely study, but the results presented are not yet fully convincing. The main observation of the difference between monocytes and hMDM in sensing Toxin B is interesting but the molecular mechanism proposed is not clear. In addition, the results should include statistics for most panels and the discussion might be shortened.

- We have added statistics to our figures for all primary data.

Major points: 

1-	It is not clear if the DSM28645 and DSM29688 strains used are isogenic? if not the authors should mention that the data shown Fig 1A are correlative and might be TcdA, TcdB dependent.

- We thank the reviewer for highlighting this point. The strains are not isogenic (it was written otherwise due to a miscommunication between authors) and so we have updated the text (Line 198) to indicate this, and that the data is correlative.

2-	The finding that the TcdB activity is not required for the NLRP3 activation suggest it is recognized as a PAMP. It would be interesting to determine the minimal motif that would be sensed by NLRP3, and this motif should not be in TcdA. Expression of TcdB truncation in a reconstituted NLRP3 inflammasome in 293T (artificial but useful) model could be an option or in BLaER1 or THP-1. Another hypothesis is the activation of NLRP3 via a TcdB specific membrane receptor. In this receptor hypothesis, transfection of both TcdB or the TcdB NXN should not activate NLRP3.

- We agree that investigating how the toxin activates NLRP3 is an interesting question. However, there are some problems with approaching this experimentally. TcdB enters the cell through the endosomal pathway, and only the GTD is delivered into the cytoplasm. As such, expression of the different domains of the toxin into the cytoplasm is non-physiological and is likely to lead to artifacts. The alternative is to make many mutants recombinantly. However, unfortunately, it is not possible to make so many mutants recombinantly due to the difficulties in expressing them, and so the question cannot be approached from this angle either. The transfection experiment is also interesting, but we have had large issues transfecting the hMDM without causing excessive cell death. Indeed, the electroporation device we used for the siRNA is the only method we have found that enables transfection of these cells without triggering large amounts of cell death. Furthermore, the toxin requires an acidic pH to alter its conformation and expose the GT domain, and that specific phosphotinositides to activate the protease domain, neither of which can occur if it is electroporated into the cytoplasm. Therefore, we have no control for knowing whether the toxin has entered the cell or not, and a negative result is inconclusive. Given the issue assessing this question, and that the focus of this study is the regulation of the Pyrin inflammasome, we believe that this requires a different study to examine properly.

3-	To strengthen their conclusion the authors should investigate other RhoGTPase inhibiting toxins such as C3 or TecA that were shown to activate Pyrin through RhoA inhibition and determine if Pyrin or NLRP3 are required in this context.

- To attempt to address this point we have added new data to the manuscript in Fig S1F where we stimulate monocytes or macrophages with the Pyrin-activating bile acid derivative characterised in “Alimov I. et. al. “Bile acid analogues are activators of Pyrin inflammasome” JBC 294(10) 2019”. Similar to the toxin derived data, we found that this bile acid analogue triggered an inflammasome response in monocytes but not hMDM, and that this is inhibited by colchicine and VX-765 but not CP-456,773, indicating it is a Pyrin response. Furthermore, prolonged priming of hMDM with LPS enables a colchicine sensitive inflammasome response to the bile acid analogue, demonstrating that prolonged LPS priming enables a Pyrin response to this trigger as well. Whilst we acknowledge that this does not directly test the relevance of other toxins, we are unable to use the C3 toxin with its physiological delivery mechanism as we could not obtain this, and would be unable to use test it in our laboratory due to restrictions on its use. We were also unable to source TecA. However, we would highlight our results using TcdA, which does not trigger an inflammasome response in human macrophages, suggesting that Pyrin is not active in these cells. We would also highlight that TcdB-mediated NLRP3 activation is entirely independent of RhoA inactivation, suggesting that RhoA is not relevant to this response.

4-	In Figure S1A, the IL-1b secretion is only partially toxin dependent and only partially NLRP3 dependent and IL-18 is increased in TcdA/B-? this is confusing compared to the Fig1A and the results with the supernatant. Only 3 points are visible in the graph. Is this statistically relevant or more replicates are needed to conclude?

- We agree with the reviewer that this data is not sufficiently conclusive to use as it was presented. As such, we have instead removed the data and updated the text to reflect that we only looked at the effect of secreted factors from *C. diff* on inflammasome activation.

5-	It is not clear if the authors are using ASC-BFP transduced WT BLaER1 cells as indicated in the fig legend of Fig2B or BLaER1 cell line overexpressing ASC-mCherry as indicated in the main text.

- We apologise for this error and have adjusted the text to be correct, which is that they were transduced with ASC-mCherry.

6-	The figure 2C, 2D, 5F should mention the fact that cells used are NLRP3-Casp4 KO

- We have adjusted the text to include this information

7-	In the Figure 2D and 2F, why the authors analyzed the TNFa only in LPS treated cells and not with all the sample shown for IL-1b, they should at least measure it for samples with TcdB that is the focus of the paper?

- TNFa is included as a readout as it acts as a surrogate for examining whether the LPS mediated “priming signal” upstream of NLRP3 activation has been affected by the knock outs and reconstitutions used. It also serves as a control to demonstrate that the effects on inflammasome observed between the cell lines being compared is specific to inflammasome activation and not a general issue with the cells. We have now also assessed the impact of TcdB and TcdB NXN on inflammasome activation in primary hMDM to demonstrate that, in the time frame of our experiments, TcdB has no impact on TNFa secretion.

8-	A TNFa measurement in Fig S2E would allow to determine if the difference observed between Tcdb and Tcdb NxN mutant is specific to the NLRP3 inflammasome in hMDM

- We have performed the experiment and included the results in Fig S2e.

9-	A complementation in mefv ko (BLaER1 or THP1) of the human and murine forms of Pyrin could provide information.

- We agree with the reviewers and have included new data in Figure 6 answering this question. We generated Pyrin KO THP1 cells expressing either human Pyrin or murine Pyrin mutation and assessed the inflammasome response to either TcdA or the bile acid analogue (data included in figure 6B-6G). We have also tested Pyrin activation in monocytes or macrophages isolated or derived from FMF patients, which have mutations in the B30.2 domain of the Pyrin protein and compared them to non-FMF controls. This data is also included in Figure 6.

10-	In Figure S2D, the authors statement: "Furthermore, the different KO lines secreted similar amounts of TNFa in response to LPS" is rather confusing without statistics.

- We apologies, this was not phrased correctly. TNFa was measured to ensure that the absence of an inflammasome response was not due to problems with the cell line generally. We have updated the manuscript to reflect this. The new phrasing is “Furthermore, the different KO lines all secreted TNFα in response to LPS, indicating that the loss of response was not due to a lack of response to LPS.

11-	The main hypothesis of the difference in terms of Pyrin response rely on the expression level of Pyrin in hMDM versus Monocytes. The authors should show a comparison between these 2 cell types in terms of Pyrin expression level after 18h of LPS (as in Fig 6). This is important considering that at 3h LPS Pyrin levels appears to be similar in both cell types in Fig 1C.

- We apologise for our ambiguity in our main hypotheses. Our hypotheses are that Pyrin is expressed at similar levels in monocytes and macrophages (unprimed or after 3h LPS priming), but only monocytes respond. Our main comparison is then within the hMDM to demonstrate that the Pyrin response only occurs when Pyrin expression is increased.

12-	The discussion mentioned post-translational modifications that could explain similar level of protein but different activities. This could be tested using Pyrin IP followed by WB using phospho-antibodies, anti-Ub-antibodies, 2D gels or mass spec analysis.

- We agree with the reviewer that these experiments would be interesting to investigate, but we believe they require sufficiently in-depth analysis and additional work as to constitute a new study. Such experiments require establishing a robust endogenous IP for Pyrin. We attempted to establish this to address this comment with the antibody we have, and with the only antibody used in the literature to IP Pyrin (An endogenous IP of Pyrin was only performed once ref: Papin et al. Cell death and differentiation 2007 Aug;14(8):1457-66). However, our antibody did not work for IP, and we were unable to replicate the results of this study.

Minor points :
1-	The introduction should mention the previous work on the NLRP3 sensing of RhoGTPase targeting toxins.

- A comment has been added to the introduction to mention this work.

2-	The Rac WB shown in Sup Fig-1 indicated the activity of both toxins. The actin blot is missing in Sup Fig1b monocytes and in hMDM the actin is not visible, and the Rac1/2/3 blot is missing.

- We have replaced the blots with updated immunoblots containing the Rac clone 102, Rac1/2/3 and actin.

3-	In Figure 1E the actin loading control is missing.

- Due to the cell death effects of inflammasome activation it is unfortunately not practical to include an actin control, as actin will also be reduced by inflammasome-mediated cell death. It is common practice in the inflammasome field to instead include IL-1b and caspase-1 in both supernatants and cell lysate, as we have shown here.

4-	P 18 line 431 this sentence is not clear concerning IL-1β « Notably, only LPS increased Pyrin expression after 5h, while LPS, IFN-β and IFN-γ but not TNFa, IL-1β or Pam3CS4K increased expression after 18h (Fig. 4a) »

- The sentence has been updated to remove the ambiguity.

5-	Unless I missed it, the title of Fig 6 refers to a disrupted signal in FMF mutations that is not shown "The B30.2 domain regulates Pyrin activation in human macrophages, and is disrupted by FMF mutations"

- We apologise for this. We have now included data on Pyrin inflammasome activation in hMDM derived from either control or FMF patients.

**Reviewer #3:**

In this study Mangan et al. investigate the inflammasome response to C. difficile toxins TcdA and B in human monocyte-derived macrophages (hMDM). The authors find that in contrast to monocytes, TcdA and TcdB do not signal through the Pyrin inflammasome in hMDM. Rather only TcdB, not TcdA activates the NLRP3 inflammasome in these cells. This latter aspect is not entirely novel given prior literature showing that TcdA/B activates NLRP3 in differentiated THP-1 macrophages however the difference observed between the two toxins in hMDM is intriguing. The authors propose that prolonged (18h) exposure to LPS or type 1 IFN increases Pyrin expression in hMDMs and reactivates engagement of the Pyrin inflammasome in these cells. Strengths of the study include a well-written and well-discussed paper, and the different macrophage model systems employed including primary cells and cell lines. Weaknesses are a striking lack of mechanistic insight relating to the central
findings of the paper, especially - 1) how Pyrin is held in an inactive state when monocytes differentiate to macrophages despite no change in actual expression of Pyrin between these cell types, 2) how priming relieves this inhibition and makes Pyrin conducive to being activated, and 3) how TcdB activates NLRP3 in hMDM, although on this point the authors have tried to rule out the conventional mechanisms by which TcdB works to activate the Pyrin inflammasome. There are also some technical problems with blots, images and missing data. The basic finding of the paper is interesting however there are several issues that need to be addressed.

- We thank the reviewer for the comments, and would like to clarify a few points that the reviewer highlighted.
  - The reviewer mentioned that there is prior literature showing that TcdA/B activates NLRP3 in differentiated THP-1 macrophages. However, this study did not provide genetic proof that NLRP3 was the responding sensor, and did not show data using primary cells, only relying on cell lines. Given that, in the same study, the inflammasome response to TcdB in NLRP3 KO BMDM was largely unaffected, we suggest that this study did not provide the clear evidence we do to demonstrate that the inflammasome response to TcdB is NLRP3 dependent. They also do not address or demonstrate the regulation of the Pyrin inflammasome.
  - We agree that we do not address all the mechanistic issues raised by our study. Delineating how TcdB activates NLRP3 lies outside the scope of this study, which focuses instead on the regulation of Pyrin. Whilst we do not fully delineate the mechanism holding Pyrin inactivate in hMDM under normal conditions, we demonstrate how this mechanism is overcome by transcriptional upregulation, a key method for controlling inflammatory responses in the cell. Following the comments by all reviewers, we also now demonstrate that the C-terminal B30.2 domain is likely required to prevent Pyrin responding in hMDM. Furthermore, we show that mutations in the B30.2 domain that cause Familial Mediterranean Fever enable Pyrin activation in hMDM in the absence of priming.

Major comments

1.	Is K+ efflux required for NLRP3 activation by TcdB in hMDM? This is a common mechanism of NLRP3 activation by toxins and is easily tested in presence of increasing concentrations of K+.

We appreciate the reviewers point and agree that investigating how the toxin activates NLRP3 is an interesting question. However, NLRP3 activators have been shown to be both potassium efflux dependent and independent, and so this experiment will not help us determine whether the response is NLRP3 dependent compared to CP-456,773, which inhibits NLRP3 by all activators tested so far. As the focus of this study is the regulation of the Pyrin inflammasome and, given the complexity of NLRP3 activation, we have investigated NLRP3 activation by TcdB in a different study.

2.	It is appreciated that the authors have shown independent donors on graphs, however given the spread of the data statistics are needed throughout the paper to be able to better appreciate which differences are significant and which aren't. Fig. 5b, 5e, S2a are some examples of where this is especially difficult.

- We have updated all data from experiments performed in primary cells to include statistical analysis.

3.	Fig. S1a: Unlike IL-1b, there is no IL-18 with the TcdA+/B+ strain so IL-18 produced upon infection with C. difficile not seem to be dependent on TcdA/B. However the text (lines 173-174) claims that cytokine  release was "primarily reliant on toxin expression when cells were infected directly". This needs to be explained / clarified. The requirement of a LPS priming signal for IL-18 which is thought to be pre-formed is also puzzling.

- We agree with the reviewer that this data is not sufficiently conclusive to use as it was presented. As such, we have instead removed the data and updated the text to reflect that we only looked at the effect of secreted factors from *C. diff* on inflammasome activation.

4.	Please show better blots and loading controls. a)	Fig. S1B - In the blots on the left, missing band with Rac1/2/3 polyclonal (presumably a loading control) in the TcdB lane makes it difficult to appreciate if lack of  a band in the upper blot is due to glucosylation or a loading issue. Similarly b-actin loading control in the blots on the right is hardly visible. b)	Fig. 1E - Loading controls on cells lysates are needed alongside the IL-1b and caspase-1 blots.

- We have replaced the blots with updated immunoblots containing the Rac clone 102, Rac1/2/3 and actin.
- Due to the cell death induced by inflammasome activation it is unfortunately not practical to include an actin control, as the actin signal will also decrease in the event of inflammasome mediated cell death. It is common practice in the inflammasome field to instead include IL-1b and caspase-1 in both supernatants and cell lysate, as we have shown here.

5.	Fig. S1C: Disrupted actin is not clear or visible in most images. Better resolution / higher magnification images and insets are needed to convincingly appreciate the impact of the toxin on actin disruption.

- We thank the reviewer for highlighting this and apologise for the state of the images in the figure – the image was down-sampled when uploaded for submission and we did not catch the change in quality that occurred. We have increased the quality of the image and increased the intensity level of the actin staining to make the interpretation of the images possible.

6.	Fig 2a: Baseline caspase-1 activity in BLaER1 cells is quite high. From the data presented, it seems likely that the differences in caspase-1 activity seen with TcdB in presence and absence of the NLRP3 inhibitor will likely not hold when these baselines are accounted for. A blot for caspase-1 is needed to convincingly show if TcdB mediated caspase-1 activation in these cells is sensitive to NLRP3 inhibition.

- We appreciate the reviewer’s comment on this, but we do not think that the caspase-1 activity seen in the unstimulated cells represents baseline activity but rather the background of the specific assay, which relies on a luminescent substrate that likely can be cleaved by other proteases. This is why we have included CP-456,773 in all conditions, to determine what proportion of the signal is due to inflammasome activation. Indeed, the NLRP3 inhibitor CP-456,773 reduces the caspase-1 activity in stimulated cells to the level of LPS. We have performed statistics on the experiment and found that the NLRP3 inhibitor significantly reduces the TcdB-mediated caspase-1 activity. We also highlight that the caspase-1 data does not stand alone, as both IL-1b and ASC specking also increase with TcdB treatment and are inhibited by the NLRP3 inhibitor.

7.	It is rather difficult to see ASC specks in TcdB treated cells in Fig. 2b. Higher magnification images and insets are needed in this figure.

- We have updated the images to increase their quality.

8.	To conclude that caspase-4 is not required for the TcdB response the authors would need to compare WT and Caspase-4 KO BLaER1 cells side by side in the same experiment (Fig S2a).

- We apologies for the overstatement regarding this data. We have now amended the text to say that caspase-4 deficiency does not account for the loss of the response to TcdB observed in the NLRP3 caspase-4 double KOs, as the response to TcdB is present in the caspase-4 KO but not the NLRP3-caspase-4 double KO. – currently line 410

9.	There seem to be differences in TNF responses between the different THP1 lines in Fig S2d contrary to what is stated in the text. Please explain. Statistics are needed to test if these differences are indeed significant.

- We apologise, that this was not phrased correctly. TNFa was measured to ensure that the absence of an inflammasome response was not due to problems with the cell line generally. We have updated the manuscript to reflect this. The new phrasing is “Furthermore, the different KO lines all secreted TNFα in response to LPS, indicating that the loss of response was not due to a lack of response to LPS.

10.	Fig. 3 has no corroborating data showing that Pyrin is the sensor that responds to TcdB in mouse BMDMs under the experimental conditions used by the authors. Although I appreciate that this has been previously reported, some form of data (preferably using WT and Pyrin KO BMDMs) which shows that this holds up for the authors would strengthen the key message of this figure.

- We apologise for the incorrect title of the figure legend and have changed this to say that the response to TcdB in murine cells is NLRP3-independent rather than Pyrin dependent. Unfortunately, we were unable to get access to BMDM from Pyrin deficient mice and so could not do the experiment. However, given that we observe (as others have) an inflammasome response to both TcdB and TcdA in BMDM, and that it is NLRP3-independent, it is likely due to the Pyrin inflammasome. We have also altered the text to reflect this.

11.	Figs. 3c and 3d - please show better blots and perform loading controls on the cell lysates. The TcdB bands are too dim to fully appreciate differences. In 3d, active caspase-1 in response to dA:dT appears to be less in presence of NLRP3 inhibitor. Without loading controls it is quite difficult to appreciate what is real and what is an artifact.

- To address this comment, we have repeated the experiment and immunoblot to obtain higher quality data. To make the comparisons easier, we have run the lysates or supernatants from the different conditions next to each other. We have also assessed Il-1b cleavage as well to support the caspase-1 data, as its greater expression and more efficient release enable more robust detection that that of caspase-1.

12.	The text and figure legend mentions that IL-b was also assessed in Figs 3c and 3d, however IL-1b data is missing from these figures.

- We appreciate the reviewer highlighting this error, and have now included the IL-1b data alongside the capase-1 data.

13.	The data presented do not convince me that expression of Pyrin is the limiting factor for its activation in hMDM. The terminology "necessary and sufficient" is a bit of an overstatement in this regard. Expression of Pyrin protein in monocytes and hMDM is equivalent (Fig. 1c) but hMDM still do not trigger a Pyrin response in the absence of prolonged priming. Because all experiments in Fig. 5 are performed under the cover a LPS or IFN-b priming signal, one likely possibility is that a post-translational modification induced by priming is the trigger for Pyrin reactivation. Alternatively, it is possible that a decrease in NLRP3 upon prolonged priming of hMDM reveals a role for Pyrin in these cells. This latter possibility needs to be tested and the authors should reword to text to discuss these alternatives.

- We would highlight our data showing that preventing an increase in Pyrin expression prevented a Pyrin response in the hMDM, suggesting that the increase in expression is at least necessary. To further support our hypothesis, we have also performed an experiment to demonstrate that Pyrin inflammasome activation in the Pyrin-expressing NLRP3-caspase-4 double KO BLaER1 cells is unchanged by LPS priming (Fig 5H). We have used LDH release as a readout, as it does not require transcription or prior TLR engagement. We have also provided evidence of Pyrin overexpression by immunoblot (Fig 5F). Furthermore, we also assessed Pyrin activation in Pyrin KO THP1s reconstituted with Pyrin and show that increased Pyrin expression enables a response to TcdA that also activates Pyrin (Fig 6b). However, we understand that this work was carried out in cell lines and so have rephrased the title of figure 5 to “Increased Pyrin expression is required for Pyrin reactivation in human macrophages” to remove any insinuation that expression is sufficient and adjusted the text throughout the manuscript to reflect this.
- In answer to the comment “Alternatively, it is possible that a decrease in NLRP3 upon prolonged priming of hMDM reveals a role for Pyrin in these cells.” We would refer the reviewer to Fig 5B, where we assess NLRP3 activation with Nigericin at 3h and 18h post LPS and show that it can be activated at both time points – indeed activation is slightly increased after 18h. We have now included data assessing NLRP3 expression in hMDM at both time points and show that NLRP3 does not change between 3h and 18h priming with LPS (Fig S3b). We would also refer the reviewer to Fig 1B, fig S1G and fig 5A, where we demonstrate that the hMDM respond to TcdA and the bile acid analogue BAA-473 (which only activate Pyrin) after 18h LPS incubation but not after 3h. This demonstrates that Pyrin is not active in these cells in the absence of prolonged priming, and so it is not a predominant NLRP3 response that blocks or masks the Pyrin response at earlier time points. The demonstration that NLRP3 can be activated by nigericin discounts the idea that NLRP3 is in general inhibited. An alternative could be that prolonged LPS exposure specifically inhibits NLRP3 activation by TcdB. We have updated the manuscript to include this comment in the discussion (line 908).

14.	Related to the above comment, does NLRP3 expression go down upon prolonged priming (18h) thereby making Pyrin the preferred sensor in hMDM? The authors should assess NLRP3 expression at 5 and 18h (Fig. 4). This is important because NLRP3 expression is also induced by LPS and some of the other stimuli tested by the authors.

- We have performed this experiment and included this data, though to be in line with the inflammasome stimulation experiments, we have assessed expression at 3h and 18h (Fig S3b). However, we also highlight our result shown in Fig 5B. NLRP3 activation is complex and requires post-translational modification, which can inhibit or enhance NLRP3 activation in the absence of changes in NLRP3 protein. Therefore, we originally elected to assess NLRP3 activity at 18h, rather than expression. As can be observed, the NLRP3 response to nigericin in hMDM after 18h is essentially unalterered (except for one, donor, where the response is increased), demonstrating that NLPR3 can be activated at 18h as efficiently as at 3h.

15.	Fig 5E - The decrease in IL-1b in TcdA treated cells in presence of the TRIF inhibitor is not convincing and seems modest at best. Is this difference significant?

- The difference is significant, but this may not have been evident from the way the data was displayed, as TcdA is considerably less effective at activating Pyrin than nigericin is at activating NLRP3. We have now separated the TcdA and nigericin data into separate graphs to enable easier visualisation of the data and performed statistical analysis.

16.	Fig 6 is incomplete. The title of the figure and discussion section imply that there is data relating to the B30.2 domain and FMF mutations in this figure but these data are not present in the paper.

- We thank the reviewer for highlighting this problem. To address this question, we have included new experimental data assessing Pyrin activation in Pyrin KO THP1 cells reconstituted with either human Pyrin or murine Pyrin. We have also assessed Pyrin activation in monocytes and hMDM derived from FMF patients or healthy donors (Fig 6).
