# Supplementary material for: Transcriptional licensing is required for Pyrin inflammasome activation in human macrophages and bypassed by mutations causing familial Mediterranean fever
Source: PLoS Biol. 2022 Nov 7;20(11):e3001351. doi: 10.1371/journal.pbio.3001351 (PMC9671422; doi:10.1371/journal.pbio.3001351)
Supplement: Attachment — Submitted filename: Pyrin_review_reviewer_responses_final.docx [file pbio.3001351.s007.docx]

**Point-by-point response (PBIOLOGY-D-21-01608R1)**

We thank the editor and reviewers for their thoughtful review and constructive comments, which helped us to revise and improve our manuscript. We have now added new data using more Familial Mediterranean Fever (FMF) patient material containing the disease-causing M6694V and M680I mutations to support our previous data. The reviewers’ comments were carefully considered and addressed below, and changes in the text prompted by the reviewers’ comments are in red.

Together, our previous and new supportive findings have strengthened the revised manuscript

which support the following concepts:

- **Mutations in the B30.2 domain that cause Familial Mediterranean Fever enable Pyrin inflammasome activation by TcdA in human macrophages and a mixed Pyrin/NLRP3 response to TcdB.**

**Reviewers comments:**

Reviewer #1: 

The authors have strengthened their data by including more healthy controls, by checking the specificity of the pyrin antibody, adding another pyrin stimulus (BAA473) and completing the missing part on FMF patients.
While I am still convinced that this is an important story for the field, the part on FMF patient is not robust enough at this stage to be presented (at least in regards to the third panel related to TcdB). The experiments are presented with 2 to 3 patients which would fine if the patients were typical FMF patients (in terms of MEFV mutations). Yet, some of the patients have very peculiar genotypes (controversial in terms of pathogenicity) precluding to draw any conclusions on FMF patients (see major point below). 

All the other points are minor points to improve the details of the manuscript. 
I agree with the authors that the mechanisms underlying the recognition of TcdB by the NLRP3 inflammasome is out of the scope of the current manuscript (although super interesting).

Major point:
-The FMF data are impressive and very striking for the first 2 panels of Fig. 7D despite the use of only 2 to 3 patients. The data presenting in Fig 7D (panel 1 and 2) is crystal clear in regards 
-to the colchicine-independence of TcdA in FMF monocytes (Van Gorp et al)
-to the response to TcdA in hMDM from FMF monocytes (Shiba et al) (Honda et al)
-to the colchicine-dependence of TcdA in hMDM from FMF patients (Shiba et al)(Honda et al).

Unfortunately, this is not the case for the third panel of Fig. 7D (the TcdB experiment in hMDM) which is also performed with only two FMF patients, the first one compound heterozygous for E148Q/A744S, the second one compound heterozygous for M694V/A744S. The very peculiar genotypes of these patients may explain why macrophages from these 2 FMF patients behave as macrophages from HD in regards to TcdB.
Indeed, the E148Q mutation is largely recognized as a non-pathogenic mutation (see Van Gorp H et al. Ann Rheum Dis 2020, Honda Y et al J. Clin Immunol 2021). 
The A744S mutation is a variant of unknown significance whose pathogenicity is also doubtful (see Alsubaie L et al. Annals of human genetics 2019 and Honda Y et al J. Clin Immunol 2021).
In addition to the very atypical/controversial genotypes, one patient barely responded to TcdB. 
Altogether, at this stage, you can not conclude on TcdB and FMF patients based on these two patients. Other patients -preferably homozygous or compound heterozygous with two pathogenic mutations should be included. 
While repeating this experiment I suggest adding a condition where you add both colchicine and CP to see whether the NLRP3 response may hide a pyrin response. 
This is very important since at the moment, the data suggest that the TcdB response in FMF hMDM is still fully CP/NLRP3-dependent and colchicine-independent suggesting that the TcdB licensing step is still required in FMF patients hMDMs (In contrast to what stated in the abstract). This might be true but cannot be concluded with these two FMF patients with atypical mutations.
The authors should be able to rapidly obtain blood from 2-3 FMF patients with classical mutations (e.g. homozygous M694V) to finalize figure 7 and the response to TcdB in FMF patients. This is a key experiment for the present manuscript and needs to be performed to sustain the authors conclusions (or present the complexity of the pyrin infammasome regulation).

We thank the reviewer for their comments. Unfortunately, due to ethical limitations imposed by the COVID pandemic, we were unable, at the time of the initial review, to get any access to patient samples at all, then later only to those patients who were coming into the clinic anyway, limiting us to the mutations that these patients had. To answer the reviewer’s point we have now repeated the experiment with 3 more donors, two homozygous for the M684V mutation and one heterozygous for the M694V and M680I mutation. As suggested by the reviewer we have also included a condition where we stimulate in the presence of both CP-456,773 and colchicine. These are now included in figure 7. Monocytes from these donors displayed the predicted phenotype – they responded to TcdA, and this response was resistant to colchicine. The macrophages from these patients, similar to the previous experiment, respond to TcdA. Notably, these cells responded to TcdB in a partially colchicine-dependent and partially CP-456,773-dependent manner, and the response was ablated by both inhibitors used together. This demonstrates that both Pyrin and NLRP3 respond to TcdB in these cells. Given that these results differed from the two donors used originally, and that both of these donors have questionable phenotypes in relation to FMF and gave quite a weak response, we have subsequently excluded them from the analysis.

Minor points:

1-Line 140-147 (from the track change version): The pyrin-dependent signaling of TcdA/ TcdB in BMDM is well demonstrated including with genetic demonstration (see van gorp et al. PNAS 2016, Fig 1 and Fig S1 fro both TcdA and TcdB, see Fig 1& 3,4 and extended fig1 in Xu H et al Nature 2014 for TcdB

We have inserted this into the paragraph line 133

2-Line 348-349: since BlaER1 cells are typically considered as monocyte/macrophage and since you did not check whether THP-1 monocytes are pyrin-responsive, please change the Figure 3 title to "NLRP3, not pyrin, is the responding inflammasome sensor to TcdB in human macrophages BlaER1 cells and THP-1 macrophages."

Thank you for this suggestion, we agree and this has been changed

Also consistently use the terminology monocytes/macrophages (or cells) for the BlaER1 cells instead of macrophages (e.g. line 941)

We have edited the entire text for consistency and they are now called BLaER1 cells throughout the manuscript

3-Line 408: please rephrase: "these cells that they still released..."

Has been edited to “these cells still released”

4-Line 410-Fig S2c?+ correct line 429

We have altered the figure so that it aligns correctly with the text. We apologise to the reviewer for this error.

5-line 667: please edit "more pyrin than"

Has been edited

6-line 700: please correct S208 instead of S204

Has been edited

7-Line 744: please check figure numbers and letters

These have been checked and edited

8-The experiments with BAA-473 (Fig. 7B) does not make sense based on your current model. You are overexpressing pyrin hence you should have an active pyrin inflammasome in human cells. This should be discussed.

Thank you for raising this point, it is well taken. We have added a comment to the discussion commenting on this result, which was strange to us as well. It begins on line 914 and reads “Notably, unlike TcdA, BAA-473 did not activate hPyrin even when it was overexpressed in THP1 macrophages. It is unclear why this result differs from that from the primary hMDM. However, the mechanism through which BAA-473 activates Pyrin is still unknown, and so could differ between these cell types. Further research understanding the mechanism of Pyrin activation by BAA-473 may provide new data that explains this result.

9-line 750: Replace "demonstrating that it is likely" by "suggesting" and line 761 "indicated" by "suggested" (murine pyrin differs in several aspects from human pyrin including but not limited to, the lack of the B30.2 domain).

These words have been edited.

10-line 906-910. This is inaccurate see Omenetti A et al ARD 2014, Jamilloux Y et al. Rheumatology 2017, Honda Y et al J. Clin Immunol 2021

We have inserted the following statement in the text (line 944) “there has not been a context identified where FMF mutations in Pyrin lead to a clear-cut gain-of-function phenotype, where FMF mutations in Pyrin have enabled a response in a context where WT Pyrin does not respond at all. However, studies in monocytes from FMF patients show increased IL-1β secretion compared to healthy donors in response to TcdB, and respond to lower doses of TcdB than healthy donors, suggesting that the FMF mutations render Pyrin more sensitive to activation. Furthermore, monocytes from FMF patients secrete more IL-1β in response to LPS stimulation (though this was NLRP3-dependent) and some studies have suggested that Pyrin containing FMF mutations cause macrophages to be hyper-inflammatory compared to those from healthy donors.”

We have also removed the sentence referring to the van Gorp paper, as the Jamilloux paper is more detailed and relevant.

11-Line 937-938: this alternative hypothesis does not make sense since when you block pyrin with colchicine, the pyrin pathway cannot predominate and yet you still do not have NLRP3 activation.

This is a good point, we have removed the alternative hypothesis

12-Line 951; you are right it has not been tested in hMDM (although AIM2 responds to T. gondii in hMDM to activate caspase-8 Fisch D et al. EMBO 2019) but it has been tested in human BMDM (Gaidt MM et al, cell 2017), so I am not convinced that Aim2 can be used as an example to compare monocytes and macrophages (in contrast to the species specificity see lines 978).

We have removed the relevant text and removed “yet”

Reviewer #2: The authors greatly improved both the quality and the interest of their manuscript clarifying and adding new data including with FMF patient samples. Nevertheless, I still have 2 minor points regarding an unanswered question and the new added data. 

Minor points:
1-	Unless I missed it, I was not able to find the comment in the introduction referring to the recently described role of NLRP3 in sensing toxins and virulence factors targeting Rho GTPases.  Please provide line and page number.

We apologies for our oversight, a comment and reference is now in the text, line 93-94. It reads: “Interestingly NLRP3 is also activated by perturbation of RhoGTPase family members, as the CNF-1 toxin, which modifies and permanently activates Rac, triggers NLRP3 activation.”

2-	The new figure 7 is a major addition to the work. I would be recomend to increase the number of samples and to perform statistics.

We thank the reviewer for their comments. Unfortunately, due to ethical limitations imposed by the COVID pandemic, we were unable, at the time of the initial review, to get any access to patient samples at all, then later only limited access to those patients that were coming into the clinic anyway, limiting the number of patients available. As highlighted by reviewer 1, some of the mutations carried by these patients have been associated with FMF but the link was not as apparent as with the M694V and M680I mutations. To answer the reviewers point we have now repeated the experiment with 3 more donors, two homozygous for the M684V mutation and one heterozygous for the M694V and M680I mutation. We have also included a condition where we stimulate with TcdB in the presence of both CP-456,773 and colchicine. These are now included in figure 7. Monocytes from these donors displayed the predicted phenotype – they responded to TcdA and this response was resistant to colchicine. The macrophages from these patients, similar to the previous experiment, respond to TcdA. Notably, these cells responded to TcdB in a partially colchicine dependent and partially CP-456,773 dependent manner, and the response was ablated by both inhibitors used together. This demonstrates that both Pyrin and NLRP3 respond to TcdB in these cells. Given that these results differed from the two donors used originally, and that both of these donors have questionable phenotypes in relation to FMF and gave quite a weak response, we have subsequently excluded them from the analysis. We understand that this reduces the number of patients, however the results are clear enough that we were still able to perform statistical analysis.

Reviewer #3: Mangan et al present a revised version of their paper. The authors have adequately addressed some of my comments and furnished missing data, however issues remain. I would request the authors to pay attention to detail in their submission as many figure panels in the reviewer responses (in elsewhere in the text) are mis-cited making it quite tedious for the reader to follow the paper.

Major issues:
1. It is unclear what is being referred to as disrupted actin cytoskeleton in the images in Fig. S1D. Pointing this out with arrowheads would be useful.

We have added arrows to the images as suggested and inserted the following text into the relevance results section (line 319): “ as demonstrated by a redistribution of the Phalloidin signal. In LPS treated cells this is distributed through the cytosol, whilst treatment with the toxin leads to accumulation of Phallodin puncta and redistribution to the the edges of the cell.”

2. Fig. S1B: Unclear what the difference between the two lanes on the blots is. Please label them.

They are now labelled

3. Lines 238-239: '…found that Pyrin expression was comparable in hMDM and monocytes (Fig. 1c, Fig S1c)." This is clearly not the case. Pyrin expression in monocytes is less that in than hMDM (Fig. 1C, S1B, S1C) even though pyrin isn't being activated in hMDM. The text needs to state so.

This has been edited. The text now reads (line 291): surprisingly higher in hMDM than in monocytes, even though we found no Pyrin-dependent inflammasome response.

4. Fig. S3A: The response of both 3h and 18h IFN-beta primed cells to TcdB is reduced with colchicine. This needs to be explained / clarified, as isn't it only the 18h Pyrin mediated IL-18 that you expect to be sensitive to colchicine?

We thank the reviewer for the comment and updated the text to read (line 618): Similarly, pre-treating hMDM with IFN-β for 18h was sufficient to render TcdB-mediated IL-18 release insensitive to CP-456,773, in contrast to the response at 3h. However, TcdB-mediated IL-18 release at both 3h and 18h was inhibited by colchicine. This as in contrast to what was seen with LPS and suggests that the NLRP3 inflamamsome response can partially inhibited by colchicine in the context of pre-treatment with IFN- β. These results indicate that IFN- β could also prime a Pyrin response (Fig. S3a).

5. Fig S3 legend needs to be fixed.

This has been edited to reflect what is shown in the figure

6. Fig. 3b: please indicate which color on the images corresponds to what either in the figure or in the legend.

We have added a colour notation to the figure and added the following text into the figure legend: ASC is in blue, nuclei are red.

7. Data in Fig. 7 on the B30.2 domain of human pyrin in inhibiting its activation are inconclusive. In order to claim an inhibitory role for the B30.2 domain in activation of human pyrin the authors would need to delete this domain in human pyrin and show the data alongside full length human pyrin in Fig. 7B. A comparison with mouse pyrin is not sufficient as there could be many other factors, sequence dissimilarities etc contributing to differences in pyrin activation between these two species. The response of human pyrin without the B30.2 domain to TcdA and BAA-473 needs to be tested to reach useful conclusions.

We thank the reviewer for their comments. We agree that the results from the experiment referring to murine Pyrin are not sufficient on their own to conclusively determine the role of the B30.2 domain. However, when taken in conjunction with the data from the macrophages derived from FMF patients, which all have mutations in the B30.2 domain, we believe that there is some supporting evidence for our claim. We have also edited the text as recommended by reviewer 1 and replaced "demonstrating that it is likely" with "suggesting" and line 761 "indicated" by "suggested" to reduce the magnitude of our claim.

8. I am a bit confused by the data in Fig. 7B where the authors show that BAA-473 cannot activate human pyrin even upon its overexpression. This runs contrary to the data in human MDMs in the rest of the paper (Fig. 6A for example) where it is implied that induction of pyrin expression by prolonged exposure to LPS can lead to its subsequent activation by BAA-473.

We also found this result a bit confusing as it did not fit with the primary cell data. To address this, we have added a comment to the discussion of this result, which was strange to us as well. It begins on line 909 and reads “It is unclear why this result differs from that from the primary hMDM. However, the mechanism through which BAA-473 activates Pyrin is still unknown, and so could differ between hPyrin and mPyrin. Further research into the mechanism of Pyrin activation by BAA-473 may provide new data that explains this result.”

9. In Fig. 7D the same FMF donors/mutations are not assessed for TcdA and TcdB responses. Different mutations can act in different ways making it difficult to appreciate any conclusions drawn from the middle and right (hMDM) panels in 7D.

We thank the reviewer for their comments. Unfortunately, due to ethical limitations imposed by the COVID pandemic, we were unable, at the time of the initial review, to get any access to patient samples at all, then later only limited access to those patients that were coming into the clinic anyway, limiting the number of patients available and also the amount of material per patient. As highlighted by reviewer 1, some of the mutations carried by these patients have been associated with FMF but the link was not as apparent as with the M694V and M680I mutations. To answer the reviewers point we have now repeated the experiment with 3 more donors, two homozygous for the M684V mutation and one heterozygous for the M694V and M680I mutation as monocytes with TcdA and as macrophages with both TcdA and TcdB. This enables direct comparison with the genotypes across all stimuli used. These are now included in figure 7. Monocytes from these donors displayed the predicted phenotype – they responded to TcdA and this response was resistant to colchicine. The macrophages from these patients, similar to the previous experiment, respond to TcdA. Notably, these cells responded to TcdB in a partially colchicine dependent and partially CP-456,773 dependent manner, and the response was ablated by both inhibitors used together. This demonstrates that both Pyrin and NLRP3 respond to TcdB in these cells. Given that these results differed from the two donors originally used in the TcdB experiment, and that both of these donors have questionable phenotypes in relation to FMF and gave quite a weak response, we have subsequently excluded them from the analysis. We understand that this reduces the number of patients, however the results are clear enough that we were still able to perform statistical analysis.

10. Donor numbers for which the western blot is shown in Fig. 7E are not the same as those used for assessing functional responses in 7D. A western blot for NLRP3 is also needed alongside the pyrin and actin blots in Fig. 7E to appreciate whether or not the NLRP3 sensitive effects claimed in Fig. 7D rightmost panel are related to differences in NLRP3 expression among patients.

We thank the reviewer for the comment. The numbers on the blot corresponded to the donor numbers in table 1. We have now updated the figure to include the table in it and have relabelled and cropped the blot to only include the donors that were also tested functionally. As mentioned in the comment above, we have removed donor 4 and 5 due to the concerns raised by reviewer 1.

The Pyrin immunoblot was performed to determine if the differences we saw were driven by increased Pyrin expression. Given that the response to Nigericin is the same between healthy donors and FMF donors, indicating no real change to the NLRP3 response, we are unclear what an immunoblot of NLRP3 would add to the study. Given the mixed response to TcdB (both Pyrin and NLRP3 dependent) it might be interesting in the future to use a single cell-based technique to determine which cells respond with Pyrin or NLRP3 to TcdB. However, this is currently not possible due to a lack of FACS capable Pyrin and NLRP3 antibodies.

11. It is unclear why are FMF cells in Fig. 7D primed with P3C? Why prime these cells at all? The point being made in the discussion (line 791-794) and elsewhere in text is that these mutations enable pyrin activation in hMDM 'without any requirement for priming or overexpression.'

We pre-treated the cells with P3C as we wanted to induce expression of IL-1b in order to use this as a readout. Due to these cells coming from FMF patients, we have relatively little material to work with and so have lower number of cells per condition than we would otherwise. IL-1b release is a much more sensitive readout than LDH, which would be the alternative, and so easier to measure in this context. We have changed the text in the figure to pre-treated instead of primed in order to avoid confusion. We did not use LPS as it was shown in our other experiments to potentially have effects on Pyrin.

12. Lines 495-498: please rephrase as the language currently implies that LPS and P3C can cause an increase in IL-1b but not Pyrin. This is not correct as LPS causes an increase in Pyrin.

We thank the reviewer for highlighting this issue and have edited the text to read (line 579): demonstrating that they were functional but only LPS treatment increased Pyrin expression.

13. Line 491: IL-1b is not tested in Fig. 5a and its mention should be deleted from the text.

We apologise for this oversight and have removed it from the text.

14. Line 482: Fix the sentence. Do you mean "…whether inflammatory conditions increased the expression of Pyrin and thus potentially enable its activation"?

The text has been edited to read (line 557): expression of Pyrin and thus potentially enable its activation.

15. Line 475: change text to "may depend on Pyrin" and include the citation which showed that the response to TcdA/B in murine macrophages can depend on pyrin.

As we do not show that Pyrin is the responding sensor directly, when referring to our own results we have kept the phrase likely dependent on Pyrin. However, as per the reviewer’s suggestion, we have added the comment that the response to these toxins was Pyrin-dependent in other studies and have added the references to support this.

We have rephased the text to read: “Collectively these results show that, in our hands, the inflammasome response to both TcdA and TcdB in murine macrophages is independent of NLRP3. Given that the Pyrin inflammasome was demonstrated to be the responding sensor in other studies, the response we observe is likely dependent on Pyrin.”

16. Line 369: Wrong fig panel is cited. It should be Fig. S2C. Also fix the figure legend of Fig. S2 as it is inconsistent with the figure. Panels in this figure are wrongly cited in the text at other locations too.

The figure legend has been altered to fit with the text and the data in the figure.

17. Figure legend of Fig. S3 is inconsistent with the figure and needs to be fixed.

The figure legend has been altered to fit with the text and the data in the figure.

18. Lines 769-772: 'Whilst pyrin overexpression….' This sentence is confusing and it is unclear what point is being made so in the interest of clarity it may be best to delete it.

We thank the reviewer for their comment. This sentence was inserted in response to a comment from the last round of revision, so we have kept it by altered the text for clarity. It now reads (line 883): “Overexpression of pyrin in THP1 macrophages or BLaER1 cells was required for a Pyrin dependent inflammasome response, even though these cells already expressed Pyrin.”

19. Lines 745-747: 'Pyrin activation could be established for all stimuli by prolonged stimulation with either LPS or type I or II interferon….' This needs to be rephrased as pyrin 'activation' upon priming with type II IFN is not shown in the paper. Only an induction of pyrin expression is shown.

We thank the reviewer for highlighting this and we have removed the reference to type II interferon from the text.

20. Line 632: "However, in the experiment carried out in the Pyrin KO BLaER1 cells we did not determine….". This sentence is quite confusing and can be rephrased quite simply to indicate that the prior experiment was carried out in NLRP3 sufficient BLaER1 cells and Pyrin reconstitution in those cells was closer to baseline as compared the vast overexpression of Pyrin seen in Fig. 6F.

We thank the reviewer for highlighting this and have rephrased the sentence to read (line 700): this experiment was performed in NLRP3 sufficient BLaER1 cells and Pyrin reconstitution in those cells was closer to baseline as compared the vast overexpression of Pyrin seen in Fig. 6F.

21. Incorrect figure panels are cited at several places:
Line 729: should be Fig. 7e
Lines 717, 720 and 723: should be panel 7d
Lines 699 and 701: should be Fig. 7b
Line 691: should be Fig. 7c

We thank the reviewer for highlighting these errors and they have been fixed.

Minor issues:
Line 1153: 'Following, the cells…' fix this sentence.

Has been edited to read: Following this, the cells were incubated with 375 µl RPMI or 375 µl RPMI containing 10 ng/ml LPS for 2 h.

Line 1141: tryptophan

Has been edited

Line 1120-1122: 'The cells were then centrifuged at 800g…' Please check that there aren't inaccuracies in this sentence. It seems unusual that cells were harvested and plated in 24 well plates after centrifugation with virus + polybrene. Usually cells are not disturbed and are directly incubated after the centrifugation step to allow virus infection to proceed.

The method listed is the correct method we have used. We found that the method listed gave better transduction efficiency and viability compared to leaving the cells in the falcon after the centrifugation step.

Line 1090: Following treatment, "cells" were… Fix the sentence.

Has been edited

Line 1000: fix the sentence. Vx-765 "for" 30 min….

Has been edited

Line 982: "where" applicable

Has been edited

Lines 984-986: Fix sentences. Precoated "with" poly l lysine…; removed and "replaced with" fresh media…

Has been edited

Line 968: generate

Has been edited

Line 966: manufacturer's

Has been edited

Line 955: immunoblot

Has been edited

Fig. S1C, line 1366: four different donors should be 'two' different donors.

Has been edited to 3 different donors as three donors are shown.

Line 885: irrelevant citation

Has been removed

Line 745: steady state

Has been edited

Line 648: dephosphorylation

Has been edited

Line 616: …more Pyrin 'than' the control…

Has been edited

Line 607: 'prevent' Pyrin activation should be changed to 'decrease' or 'reduce'.

Has been changed to reduce
